# Decoupling Network Architecture and Sorption Mechanisms in Cellulose–Alginate and Carboxymethylcellulose–Alginate Hydrogels

**DOI:** 10.1021/acsomega.6c07886

**Published:** 2026-10-03

**Authors:** Paula Paulino Silva, Vinicius Augusto de Melo Gomes, Juliano Brisola, Francisnara Tonholi, Gizilene Maria de Carvalho

**Affiliations:** Department of Chemistry, 37894 State University of Londrina, Rodovia Celso Garcia Cid, Pr 445 Km 380|Campus Universitário, Londrina 86057-970, Paraná, Brazil

## Abstract

Understanding how network architecture governs the properties of polysaccharide-based hydrogels is essential for designing sustainable functional materials. This study investigates structure–property relationships in renewable cellulose/alginate (CEL/AL) and carboxymethylcellulose/alginate (CMC/AL) hydrogels using adsorption experiments as mechanistic probes of network organization, hydration behavior, and adsorbate transport. Structural analyses revealed distinct architectures: CEL/AL retained partial cellulose crystallinity and localized chain ordering despite presenting a heterogeneous porous morphology, whereas CMC/AL exhibited a predominantly amorphous structure associated with greater chain flexibility, higher carboxylate functionality and greater hydration capacity. These structural and chemical differences strongly influenced diffusion pathways and sorption behavior. Adsorption kinetics showed pseudo-second order behavior for methylene blue adsorption in both hydrogels and for Cd^2+^ adsorption in CMC/AL, while Cd^2+^ uptake in CEL/AL followed pseudo-first order kinetics. Isotherm analyses indicated Freundlich behavior for methylene blue adsorption onto CEL/AL and Langmuir/Sips behavior for Cd^2+^, whereas CMC/AL followed the Sips model for both contaminants. Dubinin–Radushkevich analysis indicated differences in the model-derived adsorption energy between CEL/AL and CMC/AL. Both hydrogels achieved removal efficiencies above 50%, highlighting their potential for water treatment. The results indicate that chemical functionality, network organization, and hydration state jointly influence sorption behavior and functional performance.

## Graphical abstract

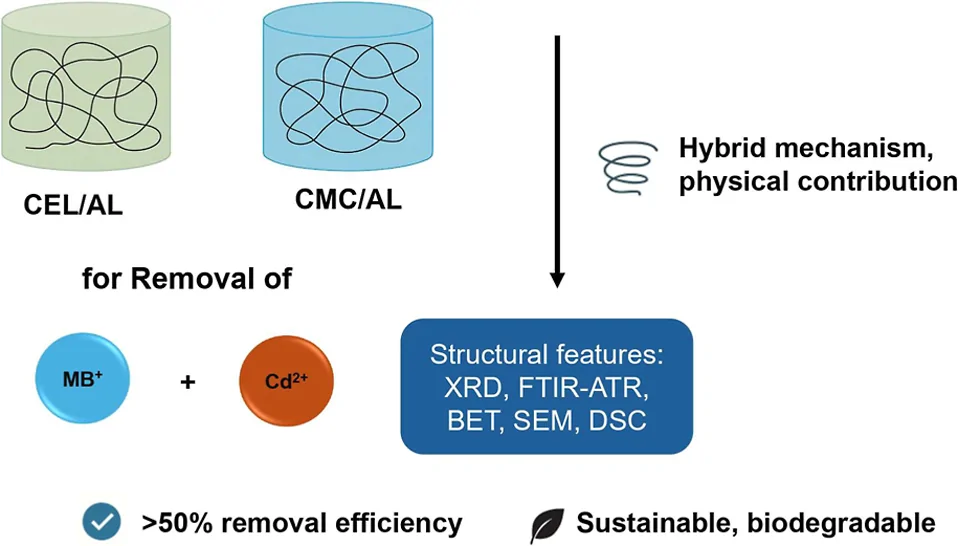

## 1 Introduction

Hydrogels based on renewable polysaccharides have emerged as versatile soft materials for environmental applications, particularly in water purification. Their appeal lies not only in the abundance, biocompatibility, and biodegradability of cellulose and alginate, but also in their ability to form hydrated, ion-responsive networks capable of interacting with a wide range of contaminants.^1^ However, despite extensive studies on polysaccharide-based hydrogels, the fundamental relationship between network architecture and functional performance remains incompletely understood, particularly regarding how crystallinity, hydration state, and polymer–polymer interactions jointly control diffusion pathways and accessibility of adsorption sites.^2^

Cellulose (CEL) and carboxymethylcellulose (CMC) provide an ideal platform to investigate these relationships due to their contrasting structural characteristics. Cellulose typically exhibits restricted chain mobility and partial recrystallization upon coagulation, which can limit swelling and reduce the accessibility of functional groups.^3^ In contrast, CMC presents higher chain flexibility, a predominantly amorphous structure, and additional carboxylate groups that enhance hydration and facilitate intermolecular interactions. When combined with alginate (AL), an anionic polysaccharide capable of forming egg-box structures via Ca^2+^ mediated cross-linking, these differences are expected to generate hydrogel networks with distinct internal organization, hydration behavior, and transport properties.^4,5^ Cellulose and alginate based hydrogels have been widely investigated for water treatment, including the removal of dyes and metal ions.^6,7^ Cellulose/alginate systems have also been specifically investigated for MB adsorption, including cellulose/alginate hydrogels and cellulose nanocrystal–alginate beads.^8,9^ Previous studies have largely emphasized adsorption performance and material design or modification.^6,9^

In many polysaccharide hydrogel systems, adsorption is not governed solely by external surface area, but by swelling-controlled, diffusion-limited mechanisms. In such systems, the penetration of adsorbates into the network is dictated by chain mobility, cross-link density, polymer–polymer interactions, and the degree of amorphousness, which together regulate both molecular diffusion and the accessibility of functional groups.^1^ Nevertheless, direct comparison of CEL/AL and CMC/AL hydrogels under equivalent formulation conditions, relating their hydration and structural characteristics to sorption behavior, remains comparatively limited.

In this context, adsorption is deliberately employed in this study as a mechanistic probe rather than solely as a performance metric, providing indirect information on network hydration and adsorbate transport.^10^ The independent adsorption of methylene blue (MB) and Cd^2+^, representing an organic cationic dye and a divalent metal ion, respectively, enables comparison of the sorption behavior of the hydrogels toward chemically distinct cationic contaminants.^11–13^ This strategy enables evaluation of how polymer–polymer interactions and structural organization are associated with sorption behavior in polysaccharide hydrogels.^14,15^

Therefore, the aim of this study is to elucidate how polymer–polymer interactions and the resulting structural organization of CEL/AL and CMC/AL hydrogels are associated with their morphological and hydration properties and with the adsorption behavior of MB and Cd^2+^ when evaluated independently. By using adsorption as a mechanistic tool to probe structure–property relationships, this work advances a structure-driven understanding of polysaccharide hydrogels and supports their development as sustainable materials for water treatment applications.

## 2 Experimental Procedures

### 2.1 Materials

Sodium alginate powder (brown algae source; purity ≥ 90%) and calcium chloride dihydrate (CaCl_2_·2H_2_O) and calcium chloride dihydrate (CaCl_2_·2H_2_O) were purchased from Dinâmica Química Contemporânea Ltda. (Brazil). Microcrystalline cellulose was obtained from Synth (Brazil). Sodium hydroxide (NaOH, purity > 99%), urea ((NH_2_)_2_CO, purity > 99%), methylene blue (C_16_H_18_ClN_3_S, *M*_w_ = 373.90 g·mol^–1^), calcium nitrate tetrahydrate (Ca­(NO_3_)_2_·4H_2_O, *M*_w_ = 236.15 g·mol^–1^), and sodium carboxymethyl cellulose (viscosity of a 1 wt % aqueous solution: 2000–3000 mPa·s; pH of 1 wt % aqueous solution at 25 °C: 6.5–8.5) were purchased from Synth (Brazil). Nitric acid (65%) was obtained from Sigma-Aldrich (Brazil), while cadmium nitrate tetrahydrate (Cd­(NO_3_)_2_·4H_2_O) was supplied by IML AB (Brazil). All reagents were of analytical grade and used as received without further purification.

#### 2.1.1 Characterization of Sodium Alginate

Prior to hydrogel preparation, the sodium alginate was characterized in terms of viscosity–average molecular weight (*M*_v_) and mannuronic-to-guluronic acid residue ratio (*M*/*G*). The viscosity–average molecular weight was determined from intrinsic viscosity measurements using the Mark–Houwink equation, employing the constants *K* = 1.5 × 10^–2^ mL g^–1^ and *a* = 0.97 reported by ABNT-NBR-7730 (1998).^16^

The structural composition of the alginate was evaluated by ^1^H NMR spectroscopy. The fractions of mannuronic acid residues (FM), guluronic acid residues (FG) were calculated from the integrated areas of the characteristic resonance signals according to the methodology described by Grasdalen (1983) and Grasdalen et al. (1979)^17,18^ makes it possible to calculate the *M*/*G* ratio and to determine, the block structure. The signals and the relative area of anomeric protons can be used for the quantitative analysis of the individual guluronic acid (FG). The area under the peak (I–III) can be used to calculate FG using the following eqs 1–3

1 $$F_{G}=\frac{A_{I}}{A_{II}+A_{III}}$$

2 $$F_{M}=1-F_{G}$$

3 $$\frac{M}{G}=\frac{F_{M}}{F_{G}}$$

Due to partial overlap of the signals and baseline noise, the peaks were individually fitted using Gaussian functions in OriginPro software before integration. The fitting procedure resulted in coefficients of determination (*R* ^2^) of approximately 0.98 for the main resonance signals and 0.94 for the GG signal.

The sodium alginate presented a viscosity–average molecular weight (*M*_v_) of 15,961 g·mol^–1^ and an *M*/*G* ratio of 0.43. Representative ^1^H NMR spectra, peak assignments, and Gaussian fitting procedures are provided in the Supporting Information (Figure S1).

### 2.2 Preparation of CEL/AL and CMC/AL Hydrogels

Hydrogels were prepared by the ionotropic gelation method, using CaCl_2_ as the cross-linking agent. Briefly, alginate (AL) and CMC were solubilized, in separated containers, in distilled water (3% and 1% w/v respectively) under magnetic stirring (300 rpm) at room temperature for 12 h. A cellulose mass corresponding to a nominal concentration of 3% was added to the NaOH/urea/water solvent system. After the dissolution procedure, a clear and homogeneous solution was obtained, with no macroscopically visible undissolved material or solid residue after centrifugation.^19^ The mixture was then frozen at −12 °C for 12 h and subsequently thawed and stirred for 30 min to form a clear cellulose solution. Then, the cellulose solution was centrifuged at 5000 rpm for 15 min to remove air bubbles.

Volumes of the corresponding polymer solutions were mixed to obtain CEL/AL ratios of 25/75, 40/60, 50/50, 60/40, and 75/25 (% w/w) and CMC/AL ratios of 25/75, 40/60, and 50/50 (% w/w), and the mixtures were maintained under magnetic stirring for 30 min. Then, the resulting solution was carefully added dropwise, with the aid of a peristaltic pump (MILAN BP600, speed 25 mL min^–1^) equipped with a tip (internal diameter of 2.0 mm), into CaCl_2_ 1% (w/v) solution (500 mL), which was kept under stirring (100 rpm) to form spherical-like beads. The as-formed beads were left to harden in CaCl_2_ solution for 24 h at room temperature. Next, the beads were thoroughly washed with distilled water to eliminate the excess of ions and then, dried at 40 °C for 48 h. The hydrogels were stored in a desiccator with silica at 25 °C for further analysis. All bead-preparation parameters, including flow rate, tip diameter, CaCl_2_ concentration, stirring conditions, and hardening time, were kept constant for each formulation to ensure procedural reproducibility.

Bead-size distribution was not quantitatively determined; therefore, no quantitative claim regarding bead-size uniformity is made.

### 2.3 Characterization of Hydrogels

#### 2.3.1 Swelling Ratio

The gravimetric method was employed to measure the swelling ratios of the hydrogels with different feed ratios in 100 mL of distilled water at 25 °C. Samples (30 mg) were put in vials filled with distilled water and kept immersed in the swelling medium at room temperature. The swelling ratio was measured in triplicate (*n* = 3) at predetermined time intervals from 0 to 480 min, and the results were expressed as mean ± standard deviation (SD). The degree of swelling (% SW) after each time interval was calculated per eq 4.

4 $$\%\;SW=\frac{W_{S}-W_{D}}{W_{D}}\times 100$$

where *W*_S_ is the weight of swollen equilibrium sample (mg) and *W*_D_ is the weight of the dried sample (mg).

#### 2.3.2 Fourier Transform Infrared Spectroscopy Attenuated Total Reflection Mode (FTIR-ATR)

Fourier transform infrared spectra (FTIR-ATR) were recorded using a Bruker spectrophotometer, model Vertex 70, equipped with ATR accessory with crystal Ge 45°, using a spectral resolution of 4 cm^–1^ and 16 scans in the range of 4000–400 cm^–1^. After acquisition, the spectra were normalized. The O–H stretching region was analyzed from the numerical spectra after Savitzky–Golay smoothing. A local linear baseline was constructed over the 2800–3800 cm^–1^ interval and subtracted from each spectrum. The O–H band maximum (ν_max_) was verified from the corresponding zero-crossing of the first derivative, with the second derivative used to distinguish local maxima from minima. Wavenumber shifts were calculated as Δν = ν_max_,_mix_ – ν_max,polymer_. The same processing parameters were applied to all neat polymers and hydrogel compositions. The positions of the other FTIR bands discussed in the manuscript were determined directly from the corresponding spectra.

#### 2.3.3 Differential Scanning Calorimetry (DSC) Analysis

The thermal behavior of the hydrogels was examined by differential scanning calorimetry (DSC) on a Shimadzu model DSC-60 equipment. DSC analyses were performed on the dried hydrogel samples before the swelling experiments. Enthalpy values were obtained by peak integration and normalized to the initial sample mass loaded into the DSC pan. 5 mg of material was used, and the scans were carried out under heating from room temperature to 350 °C at a heating rate of 20 °C min^–1^. The measurements were performed under a nitrogen atmosphere at a flow rate of 50 mL min^–1^ at atmospheric pressure. The thermograms were analyzed using TA-60WS software. Peak temperatures (*T*_peak_) were determined from the maximum of each thermal event. The enthalpy associated with the endothermic event attributed to residual physically and weakly bound water (40 to 140 °C, approximately) was determined by integration of the peak area using a sigmoidal baseline.

#### 2.3.4 Nitrogen Physisorption by Brunauer–Emmett–Teller (BET)

Microstructural properties, such as pore size distribution and specific surface area, were determined by the nitrogen sorption method. The mathematical method used to calculate the surface area was the Brunauer–Emmett–Teller method and the pore size and volume using the Barrett, Joyner and Halenda method.^20,51^ The equipment used was the Autosorb-Quantachrome NOVA 1200e (surface area & pore size analyzer). The samples were dried at 100 °C for 4 h in an oven and degassed for 2 h at 120 °C under vacuum before being subjected to N_2_ physisorption tests.

#### 2.3.5 Determination of pH at the Point of Zero Charge (pH_PZC_)

The surface charge was measured by determining the pH at the point of zero charge (pH_PZC_) by adding 0.10 g of each hydrogel in a container with 10 mL of distilled water and in another container with 10 mL of KCl solution (1 mol L^–1^) for 24 h.^21^ After this period, the pH of the solution with distilled water and the solution with KCl was measured, with the point of zero charge being obtained by the following eq 5

5 $${pH}_{PZC}=2\times pH\left(KCl\right)-pH\left(H_{2}O\right)$$

#### 2.3.6 Scanning Electron Microscopy (SEM)

Scanning electron micrographs were obtained with a scanning electron microscope (Philips-FEI Quanta200) at an accelerating voltage of 30 kV. The surface of the samples was covered with a thin layer of gold, using a metalizer SCD 050 (Baltec-Balzares).

#### 2.3.7 X-ray Diffraction (XRD) Analysis

Wide-angle X-ray diffraction measurements (WAXD) were performed on a PANalytical model X’Pert PRO MPD diffractometer operating in Bragg–Brentano geometry with Cu Kα radiation. It was used 2θ scan range from 5° to 30° with angular step of 0.05°. The voltage and current were, respectively, 40 kV and 30 mA. The time count per point was 2.0 s. The diffraction peaks were identified using OriginPro software, and the X-ray diffraction patterns were analyzed without further mathematical processing.

#### 2.3.8 Batch Adsorption Studies

The optimal adsorbent mass was determined by comparing the removal efficiency obtained with different hydrogel masses while maintaining the other experimental conditions constant. The mass providing the highest removal efficiency was selected for the subsequent adsorption experiments.

Batch adsorption studies were carried out evaluating the amount adsorbed per mass of adsorbent and the percentage of dye and metal removal efficiency were evaluated according to eqs 6 and 7, respectively

6 $$q_{e,\exp}=\frac{\left(C_{i}-C_{e}\right)}{m}V$$

7 $$removal\;efficiency\;\left(\%\right)=\frac{\left(C_{i}-C_{e}\right)}{C_{i}}\times 100$$

where *q*_e,exp_ is the amount adsorbed per gram of adsorbent at equilibrium (mg g^–1^), *C*_i_ is the initial adsorbate concentration (mg·L^–1^), *C*_e_ is the equilibrium adsorbate concentration (mg·L^–1^), *V* is the volume of the adsorbate solution (L), *m* is the mass of adsorbent (g).

For both MB and Cd^2+^, batch adsorption experiments sequentially evaluated adsorbent mass, solution pH, initial adsorbate concentration, and contact time. The specific experimental conditions and analytical procedures for each adsorbate are described below.

##### 2.3.8.1 Dye Adsorption

MB adsorption experiments were performed using 10.0 mL of solution under stirring at 60 rpm and 25 °C. A 50 mg L^–1^ MB stock solution was used, and the solution ionic strength was adjusted with 0.10 mol L^–1^ KCl. MB concentrations were determined by UV–vis spectrophotometry (UV-260, Shimadzu) at 665 nm (λ_max_) using the calibration curve provided in Figure S2. Adsorbent masses of 0.05 and 0.10 g were evaluated at 25 mg L^–1^ MB for 8 h. The mass providing the highest removal efficiency was used in the subsequent experiments. The pH effect was evaluated from pH 4 to 10, the initial MB concentration from 5 to 30 mg L^–1^, and contact time from 5 to 480 min. The solution pH was measured before and after the adsorption experiments.

##### 2.3.8.2 Adsorption Cd^2+^

Cd^2+^ adsorption experiments were performed using 10.0 mL of solution under stirring at 15 rpm and 25 °C. Ca­(NO_3_)_2_ at 0.10 mol L^–1^ was used as the supporting electrolyte, corresponding to an ionic strength of 0.30 mol L^–1^. Cd^2+^ concentrations were determined by flame atomic absorption spectrometry (FAAS; AA-6601F, Shimadzu) at 228.80 nm using the calibration curve provided in Figure S2. Hydrogel masses ranging from 0.10 to 0.70 g were evaluated at 10 mg L^–1^ Cd^2+^ for 8 h. The pH effect was evaluated from pH 4 to 10, the initial Cd^2+^ concentration from 5 to 50 mg L^–1^, and contact time from 5 to 180 min. The solution pH was measured before and after adsorption.

##### 2.3.8.3 Kinetic and Isotherm Models

To examine the adsorption mechanism, kinetic models of pseudo-first order, pseudo-second order and intraparticle diffusion were used to fit the experimental data. These models are used to describe the adsorption of dyes, pigments, and other pollutants (heavy metals) in solid sorbents.

The kinetic models such as pseudo-first order,^22^ pseudo-second order^23^ and intraparticle diffusion model^24^ were used to understand the adsorption mechanism as a function of time of all hydrogels produced. The equation and parameters of these models are shown in Table 1. The kinetic models of pseudo-first order, pseudo-second order were employed using the nonlinear fitting method and intraparticle diffusion model was fitting using the linear method. The experimental data for adsorption isotherms were obtained by measuring the equilibrium adsorption amount of MB and Cd^2+^ at different initial dye concentrations (5 to 30 mg L^–1^ and 0.2 to 3.0 mg L^–1^). The equilibrium adsorption data were interpreted using Langmuir,^25,26^ Freundlich,^26,27^ Sips^28^ and Dubinin–Radushkevich (DR)^20,30^ isotherm models, Table 1.

**Table 1:** Kinetic and Equilibrium Models Used to Fit the Adsorption Data^a^

model	equation	reference
nonlinear pseudo-first order (PFO)	$q_{t}=q_{e}\left(1-e^{-k_{1}t}\right)$	^22^
nonlinear pseudo-second order (PSO)	$q_{t}=\frac{q_{e}^{2}k_{2}t}{1+q_{e}k_{2}t}$	^23^
intraparticle diffusion (linear)	$q_{t}=k_{df}t^{0,5}+C_{df}$	^24^
nonlinear Langmuir	$q_{e}=\left(\frac{q_{m}\times k_{l}\times C_{e}}{1+k_{l}\times C_{e}}\right)$	^25^,^26^
	$$R_{L}=\left(\frac{1}{1+k_{l}C_{i}}\right)$$	
nonlinear Freundlich	$q_{e}=k_{f}\times C_{e}^{1/n}$	^26^,^27^
nonlinear Sips	$q_{e}=\frac{q_{m}K_{S}C_{e}^{1/n_{s}}}{1+K_{S}C_{e}^{1/n_{s}}}$	^28^
Dubinin–Radushkevich (DR) (linear)	$\ln q_{e}=\ln q_{s}-\left[\beta {\left(\varepsilon \right)}^{2}\right]$	^29^,^30^
	$$\varepsilon =RT\;\ln\left(1+\frac{1}{C_{e}}\right)$$	
	$$E=\left(\frac{1}{\sqrt{2\times \beta }}\right)$$	

a *q*_*t*_: adsorbed amount of the adsorbate at time *t* (mg g^–1^); *q*_e_: equilibrium adsorption capacity estimated from the model fit (mg g^–1^); *q*_e,exp_: experimental equilibrium adsorption capacity (mg g^–1^); *q*_m_: maximum monolayer adsorption capacity (mg g^–1^); *q*_s_: theoretical saturation capacity; *t*: contact time (min); *k*_1_: pseudo-first-order rate constant (min^–1^); *k*_2_: pseudo-second-order rate constant (g mg^–1^ min^–1^); *k*_df_: intraparticle diffusion rate constant (mg g^–1^ min^–0.5^); *C*_df_: constant related to the resistance to diffusion in the boundary layer (mg g^–1^); *C*_e_: equilibrium adsorbate concentration in the solution (mg L^–1^); *C*_i_: initial adsorbate concentration (mg L^–1^); *k*_l_: Langmuir constant related to adsorbate–adsorbent affinity (L mg^–1^); *k*_f_: Freundlich constant; *n*: Freundlich exponent; *K*_S_: Sips constant; *n*_S_: Sips heterogeneity parameter; *R*_L_: Langmuir separation factor; β: D–R constant (mol^2^ kJ^–2^); ε: Polanyi potential; *R*: universal gas constant; *T*: absolute temperature (K); *E*: model-derived mean adsorption energy (kJ mol^–1^).

The most appropriate theoretical models that describe the experimental data of hydrogels/dye and hydrogels/Cd^2+^ system was selected from the determination coefficient (*R* ^2^) and RMSE.

Additionally, model-estimated equilibrium adsorption capacities *q*_e_ were compared with the experimental equilibrium adsorption capacities (*q*_e,exp_) calculated from the mass-balance equation.

## 3 Results and Discussion

### 3.1 Swelling Ratio (SW %)

The swelling behavior of hydrogels provides key insights into network architecture, hydrophilicity, and chain mobility.^31,32^ The swelling ratio (SW %) of all formulations is presented in Figure 1a,b, revealing distinct hydration profiles depending on polymer composition.

**Figure 1:**
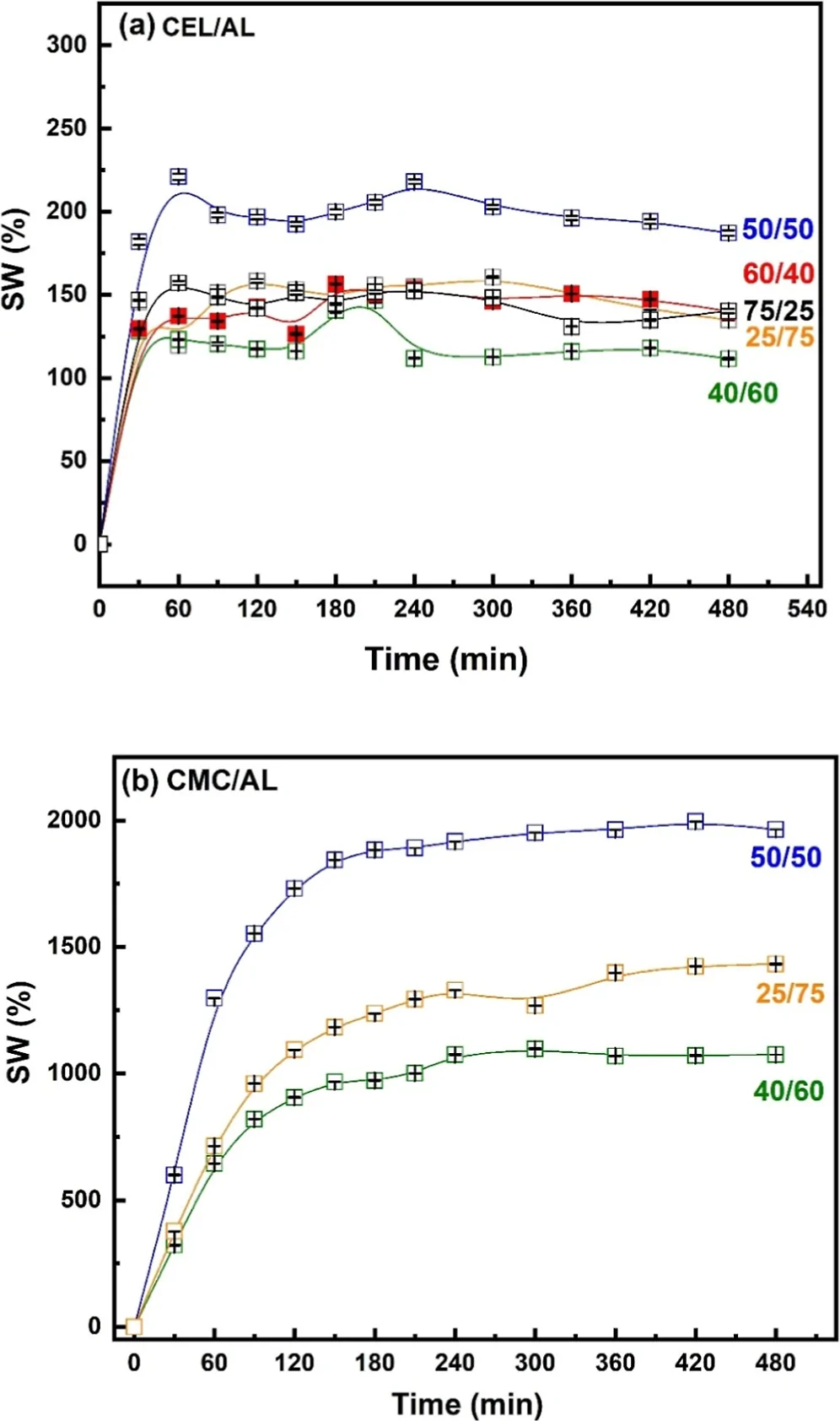
Swelling ratio (SW %) in distilled water at 25 °C of (a) CEL/AL and (b) CMC/AL hydrogels. SW % values are presented as mean ± standard deviation (SD) (*n* = 3); error bars represent SD.

In the initial stage (first 60 min), both systems exhibit a rapid increase in water uptake, which can be attributed to the high osmotic driving force and rapid hydration of hydrophilic regions within the polymer network. The interaction of water molecules with hydrophilic functional groups (−OH and COO^–^) promotes polymer chain relaxation and network expansion.

In the initial stage (first 60 min), both systems exhibit a rapid increase in water uptake, which can be attributed to the high osmotic driving force and the availability of free volume within the ionically cross-linked network. At this stage, water diffusion is facilitated by the presence of interconnected pores and the relatively low resistance imposed by the polymer chains cross-linked with Ca^2+^ ions. The interaction of water molecules with hydrophilic functional groups (−OH and COO^–^) promotes polymer chain relaxation and network expansion.

As swelling progresses, a marked reduction in the sorption rate is observed, indicating a transition from a diffusion-controlled regime to a relaxation-limited process. This behavior is associated with progressive network saturation and the establishment of polymer–water interactions, which restrict further chain mobility and hinder additional water penetration.^33,34^ Equilibrium is reached after approximately 8 h, suggesting a stable hydrogel network with defined structural constraints.

The swelling behavior also depended on the polymer ratio within each hydrogel series. For CEL/AL, the 50/50 formulation exhibited the highest SW %, whereas the remaining compositions showed lower values, with no monotonic trend as the CEL fraction increased. A similar composition-dependent behavior was observed for CMC/AL, with the 50/50 formulation also showing the highest swelling. These results indicate that swelling is not determined solely by the relative amount of either polymer, but likely reflects the combined effects of polymer composition and network organization.

The CMC/AL (50/50) hydrogel exhibits a significantly higher swelling ratio (1999%) compared to CEL/AL (50/50) (194%), highlighting the strong influence of polymer chemistry on water uptake, Figure 1a,b. This behavior is associated with the presence of carboxymethyl groups (−CH_2_COO^–^) in CMC, which enhance hydrophilicity and promote electrostatic repulsion between polymer chains, leading to increased network expansion and water accessibility.^35^ Differences in effective cross-link density may also contribute to the swelling behavior, since the additional carboxylate groups in CMC may modify polymer Ca^2+^ and polymer–polymer interactions. However, because cross-link density was not directly measured, its specific contribution cannot be independently determined. In contrast, the lower swelling capacity of CEL/AL hydrogels can be associated with the presence of crystalline domains in cellulose, which limits chain mobility and reduces the availability of accessible hydrophilic sites. The presence of crystalline domains acts as physical barriers to water diffusion, resulting in a more compact and less permeable network. These findings indicate that both chemical functionality and network organization contribute to the swelling behavior of polysaccharide-based hydrogels.^36,37^

### 3.2 Fourier Transform Infrared Spectroscopy Attenuated Total Reflection Mode (FTIR-ATR)

The hydrogels samples and the pure polymers showed similar FTIR spectra Figure 2 as well as all the characteristic bands for CEL, CMC and AL. The spectra of pure AL, CEL and CMC present a broad absorption band in the range of 3107–3647 cm^–1^, attributed to O–H stretching vibrations associated with intra- and inter-molecular hydrogen bonding. The bands observed in the range of 2756–2978 cm^–1^ correspond to symmetric and asymmetric stretching vibrations of CH_2_ and CH_3_ groups.

**Figure 2:**
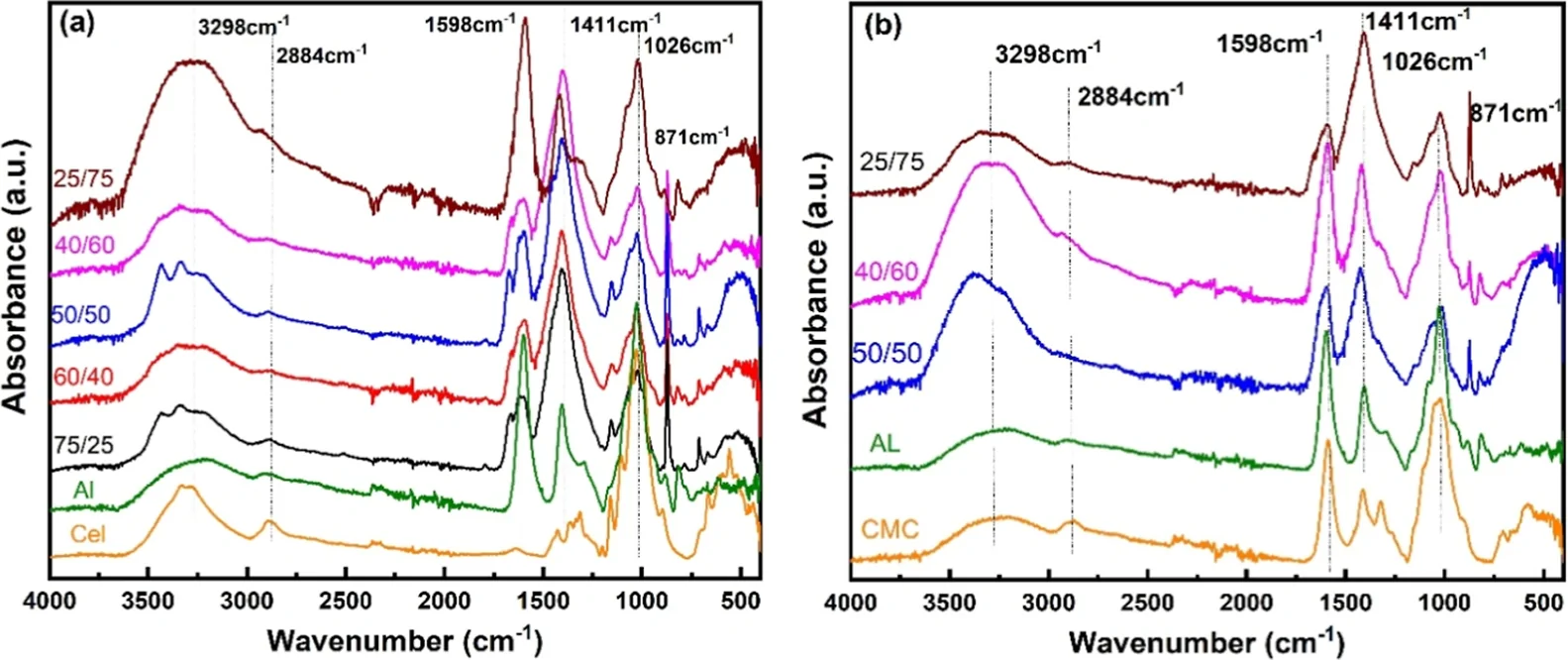
Fourier transform infrared spectroscopy attenuated total reflection mode (FTIR-ATR) spectra acquired in the range of 4000–400 cm^–1^ of: (a) cellulose, alginate and CEL/AL hydrogels and (b) CMC, alginate and CMC/AL hydrogels.

The region of 800–1200 cm^–1^ is considered as the “fingerprint” region for carbohydrates. The band at 1026 cm^–1^ is attributed to C–O–C asymmetric stretching of ring. For CMC and AL, the presence of carboxylate groups is evidenced by absorption bands in the range of 1730–1409 cm^–1^, corresponding to asymmetric and symmetric stretching vibrations of COO^–^ groups. These functional groups play a key role in the formation of ionically cross-linked networks through interactions with divalent calcium ions, contributing to the structural stability of the hydrogels.

The broad O–H stretching band was observed in the pure polymers and all hydrogel formulations.^38^ Differences in its position among the samples suggest changes in the hydrogen-bonding environment after hydrogel formation. The corresponding band positions and wavenumber shifts are summarized in Table S1.

A subtle band at 2884 cm^–1^ is observed in both systems (Figure 2a,b), corresponding to the axial deformation of methylene groups (−CH_2_−). The band at 1598 cm^–1^ is attributed to the asymmetric stretching of carboxylate groups (COO^–^). In both CEL/AL and CMC/AL hydrogels, this band exhibits broadening compared with pure alginate, indicating interactions involving carboxylate groups.^39^

This broadening can be attributed to interactions between alginate and CEL or CMC through COO^–^ groups, particularly within the mannuronic (M) blocks. This interpretation is consistent with the known structure of alginate, in which guluronic (G) blocks preferentially participate in Ca^2+^-mediated cross-linking, while mannuronic (M) blocks remain more available for intermolecular interactions.^40^ The symmetric stretching of COO^–^ groups is observed in the range of 1405–1417 cm^–1^ for CEL/AL hydrogels and 1406–1418 cm^–1^ for CMC/AL hydrogels (Figure 2a,b).^35,38,41–43^

The in-plane angular deformation of the O–H bond occurs at 1400–1300 cm^–1^ and the out-of-plane angular deformation occurs in the range of 769–650 cm^–1^. These bands are of low intensity and are situated in a region where several bands overlap, as observed in the spectrum obtained in Figure 2a,b, thereby limiting their value as elements of structural identification. A broad band at 1020 cm^–1^, assigned with the stretching of the C–O bond within the polysaccharide ring, is evident in all CEL/AL hydrogels (Figure 2a) and all CMC/AL hydrogels (Figure 2b),^44^ confirming the preservation of the polymeric structure.

Additionally, a distinct band at 872 cm^–1^ is observed in both systems and is attributed to interactions involving Ca^2+^ ions during hydrogel formation,^45^ supporting the formation of ionically cross-linked networks. Based on these observations, it can be proposed that the interactions between cellulose or carboxymethylcellulose and alginate occur predominantly within the mannuronic (M) regions, while gelation induced by CaCl_2_ is associated with guluronic (G) blocks, as illustrated in Figure 3a.

**Figure 3:**
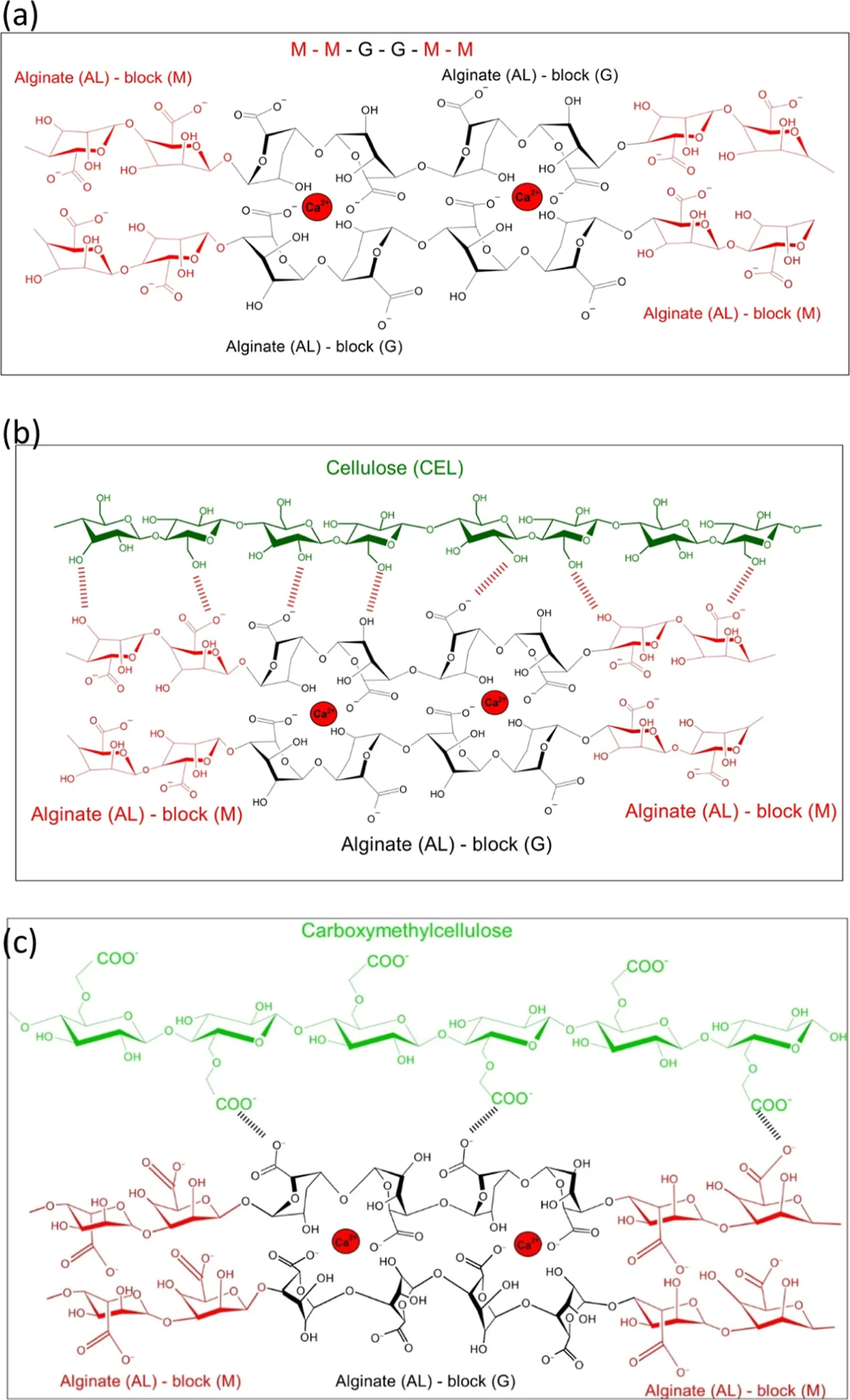
Schematic representation of the proposed interactions in (a) ionic interactions between Ca^2+^ and alginate (AL); (b) interactions between cellulose (CEL) and alginate; and (c) interactions between carboxymethylcellulose (CMC) and alginate. The schemes illustrate Ca^2+^-mediated ionic cross-linking of alginate and hydrogen-bond interactions between the polysaccharide chains. Adapted from Gomes et al. (2026),^30^ under a Creative Commons Attribution 4.0 International (CC BY 4.0) license http://creativecommons.org/licenses/by/4.0/.

CMC/AL may involve interactions through both COO^–^ and OH groups, whereas CEL/AL interactions are mainly associated with hydroxyl groups.

Additionally, a distinct band at 872 cm^–1^ is observed in both systems and is attributed to interactions involving Ca^2+^ ions during hydrogel formation,^34^ supporting the formation of ionically cross-linked networks. Based on these observations, it can be proposed that the interactions between cellulose or carboxymethylcellulose and alginate occur predominantly within the mannuronic (M) regions, while gelation induced by CaCl_2_ is associated with guluronic (G) blocks. Figure 3 is intended as a qualitative representation of the proposed interaction types and not of their number or relative strength. Accordingly, Figure 3a illustrates Ca^2+^-mediated ionic cross-linking between alginate carboxylate groups, together with hydrogen-bond interactions between CEL and AL or between CMC and AL; in the CMC/AL system, additional carboxylate groups may also participate in Ca^2+^-mediated interactions. Figure 3b shows the proposed hydrogen-bond interactions between CEL and AL, whereas Figure 3c illustrates the interactions between CMC and AL, including hydrogen bonding and the possible participation of additional CMC carboxylate groups in Ca^2+^-mediated interactions.

### 3.3 Differential Scanning Calorimetry (DSC)

Differential scanning calorimetry (DSC) was employed to investigate the thermal behavior of the hydrogels and to identify energy-related transitions associated with structural organization and intermolecular interactions within the polymeric network. The DSC thermograms of the hydrogel samples are presented in Figure 4a,b.

**Figure 4:**
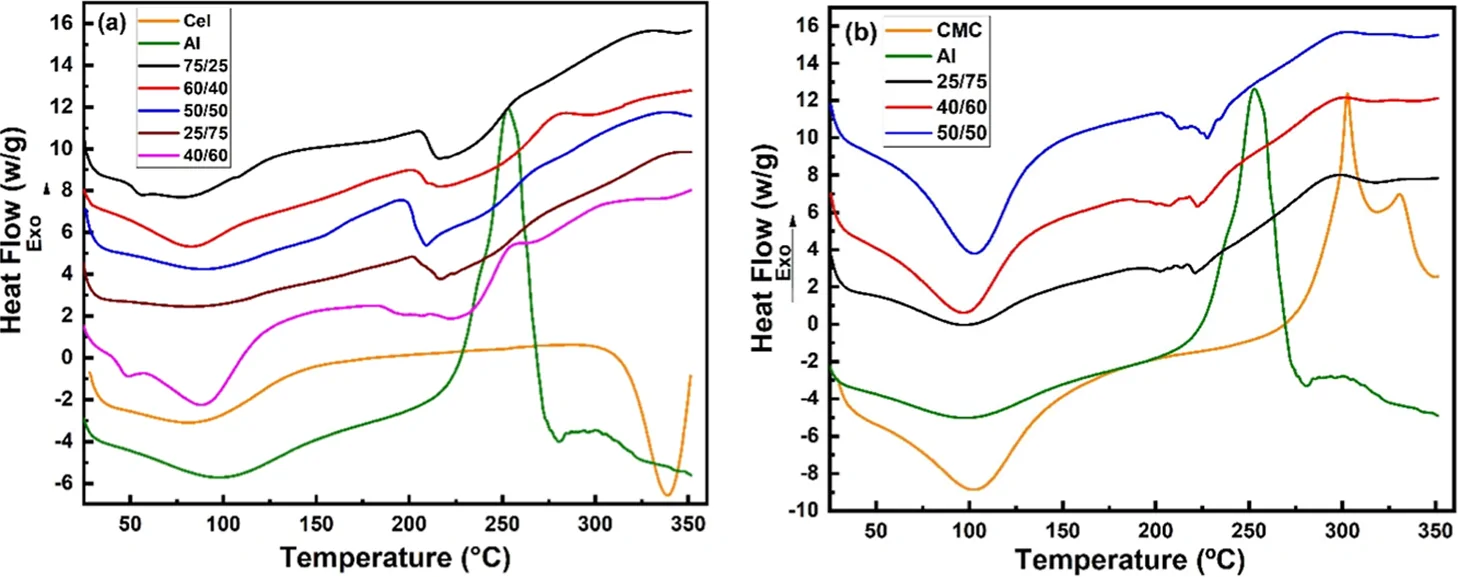
Differential scanning calorimetry (DSC) thermograms of (a) CEL, AL, and CEL/AL hydrogels and (b) CMC, AL, and CMC/AL hydrogels. Temperatures and enthalpy values associated with the thermal events are provided in Table S2.

The analyzed systems exhibited both endothermic and exothermic events, which are characteristic of polysaccharide-based materials.^46^ These thermal transitions are associated with processes such as water loss, disruption of intermolecular interactions, and structural rearrangements within the polymer network. The presence and profile of these events reflect the balance between bound water, polymer–polymer interactions, and the degree of network organization.

The first endothermic event, observed for all CEL/AL hydrogels in the temperature range of 40–140 °C (Figure 4a), is attributed to the loss of residual physically adsorbed and weakly bound water retained in the dried hydrogel network. A similar event is observed for CMC/AL hydrogels at higher temperatures (97–103 °C). This behavior reflects the enhanced hydration capacity of CMC-containing hydrogels, which is associated with the presence of carboxymethyl groups (−CH_2_COOH) that increase hydrophilicity and promote stronger polymer–water interactions. These findings are consistent with the higher swelling ratios observed for CMC/AL hydrogels, although the DSC enthalpy reflects the thermal event associated with residual water rather than directly quantifying its amount. The second endothermic event occurs in the range of 209–222 °C for CEL/AL hydrogels and 221–227 °C for CMC/AL hydrogels (Figure 4a,b), and is attributed to the degradation of glycosidic linkages within the polysaccharide backbone. This transition occurs at temperatures close to the degradation of alginate (∼253 °C), as reported in Table S2,^47^ suggesting that alginate domains contribute to the observed high-temperature thermal behavior of the hydrogel network. The third thermal event corresponds to the main degradation of the polymeric chains. For CEL/AL hydrogels, this process occurs between 255 and 283 °C (Figure 4a), while for CMC/AL hydrogels it is shifted to higher temperatures (290–300 °C) (Figure 4b), approaching the degradation temperature of CMC (∼300 °C). This shift indicates that the incorporation of CMC contributes to increased thermal stability, likely due to enhanced intermolecular interactions and a more hydrated and flexible network structure.

In the case of cellulose, the presence of extensive hydrogen bonding between chains prevents the observation of a melting transition, as thermal degradation occurs prior to melting. Therefore, the event detected at approximately 339 °C is attributed to cellulose decomposition rather than a true melting process.^48^

Based on the swelling behavior and DSC thermal profiles, the CEL/AL 50/50 and CMC/AL 50/50 hydrogels were selected for further investigation. The 50/50 composition also provides the same polysaccharide-to-alginate mass ratio in both systems, enabling their direct comparison at the same nominal composition. The adsorption experiments were designed to compare these representative formulations rather than to establish composition-dependent adsorption trends. Consequently, these systems were used in subsequent adsorption studies involving methylene blue (MB) dye and Cd^2+^ ions.

### 3.4 Morphology of Hydrogels by Scanning Electron Microscopy (SEM)

The morphology of the CEL/AL 50/50 and CMC/AL 50/50 spheres are shown in Figure 5.

**Figure 5:**
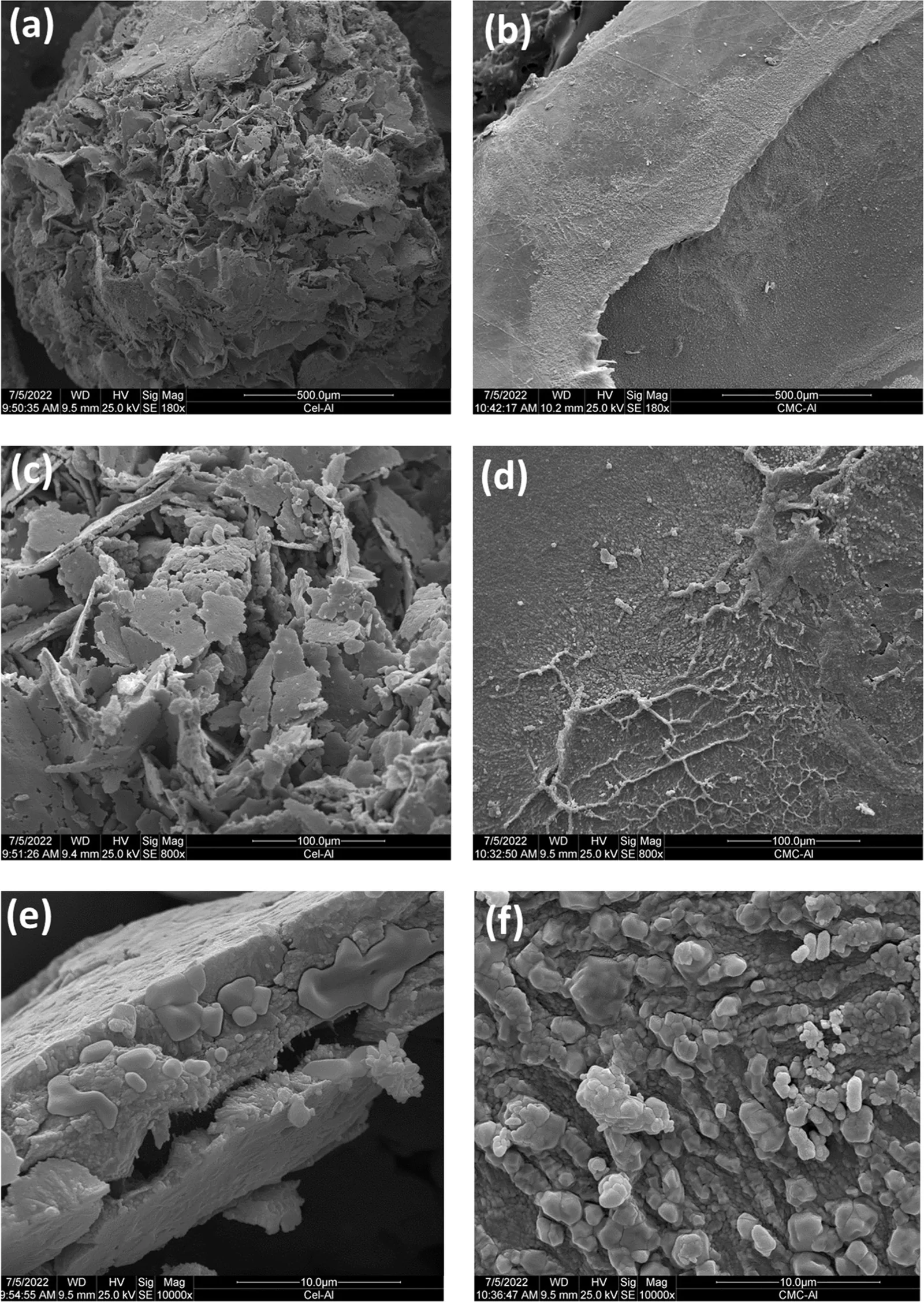
Scanning electron microscopy (SEM) micrographs of the CEL/AL 50/50 (a,c,e) and CMC/AL 50/50 (b,d,f) samples, acquired in secondary electron (SE) mode at an accelerating voltage of 25.0 kV. Images (a,b) were obtained at a magnification of 180×, with a working distance (WD) of 9.5 mm and a scale bar of 500 μm; images (c,d) at a magnification of 800×, with a WD of 9.4 mm for CEL/AL and 9.5 mm for CMC/AL, and a scale bar of 100 μm; and images (e,f) at a magnification of 10,000×, with a WD of 9.5 mm and a scale bar of 10.0 μm.

For the CEL/AL 50/50 hydrogel (Figure 5a,c,e), the beads exhibit an irregular and non-uniform spherical morphology, characterized by a rough surface with the presence of numerous voids and surface discontinuities. These structural features indicate a more heterogeneous dry-state morphology.^14^ At higher magnifications (Figure 5c,e), these voids become more evident, suggesting that the CEL-based network promotes a less compact structure. This morphology may be associated with the intrinsic structural organization of cellulose.^49^

In contrast, the CMC/AL 50/50 hydrogel (Figure 5b,d,f) presents a more compact and homogeneous surface, with fewer visible voids compared to CEL/AL. This observation is consistent with the lower specific surface area and pore volume obtained from BET analysis (Table S3, Supporting Information), supporting the formation of a denser polymeric network. This more compact morphology can be related to the interaction mechanism proposed in Figure 3 (Section 3.2), in which CMC interacts with alginate through multiple interaction sites, resulting in a more interconnected structure with reduced free volume.

According to the literature, pure alginate beads typically exhibit a rough surface;^50^ therefore, the differences observed in CMC/AL hydrogels may be associated with the influence of CMC on the structural organization of the network. Additionally, based on the average pore size values obtained for all CEL/AL and CMC/AL hydrogels (Table S3, Supporting Information), these materials can be classified as mesoporous according to IUPAC criteria.^51^ It should be noted that SEM reflects the dry-state morphology and does not directly represent the hydrogel network under swelling or adsorption conditions, where hydration may induce structural reorganization.

Although the specific surface area and porosity values obtained by nitrogen physisorption (BET) are relatively low compared to similar systems reported in the literature,^14,52,53^ the hydrogels exhibited significant contaminant removal efficiency. Because BET measurements characterize the dry-state texture, these values primarily reflect the hydrogel structure in the dry state rather than its hydrated state under aqueous adsorption conditions.

### 3.5 X-ray Diffraction

X-ray diffraction (XRD) was used to verify the structure of the material regarding crystallinity. Figure 6 shows the diffractograms of the CEL, AL, CMC polymers, and the CEL/AL 50/50 and CMC/AL 50/50 hydrogels.

**Figure 6:**
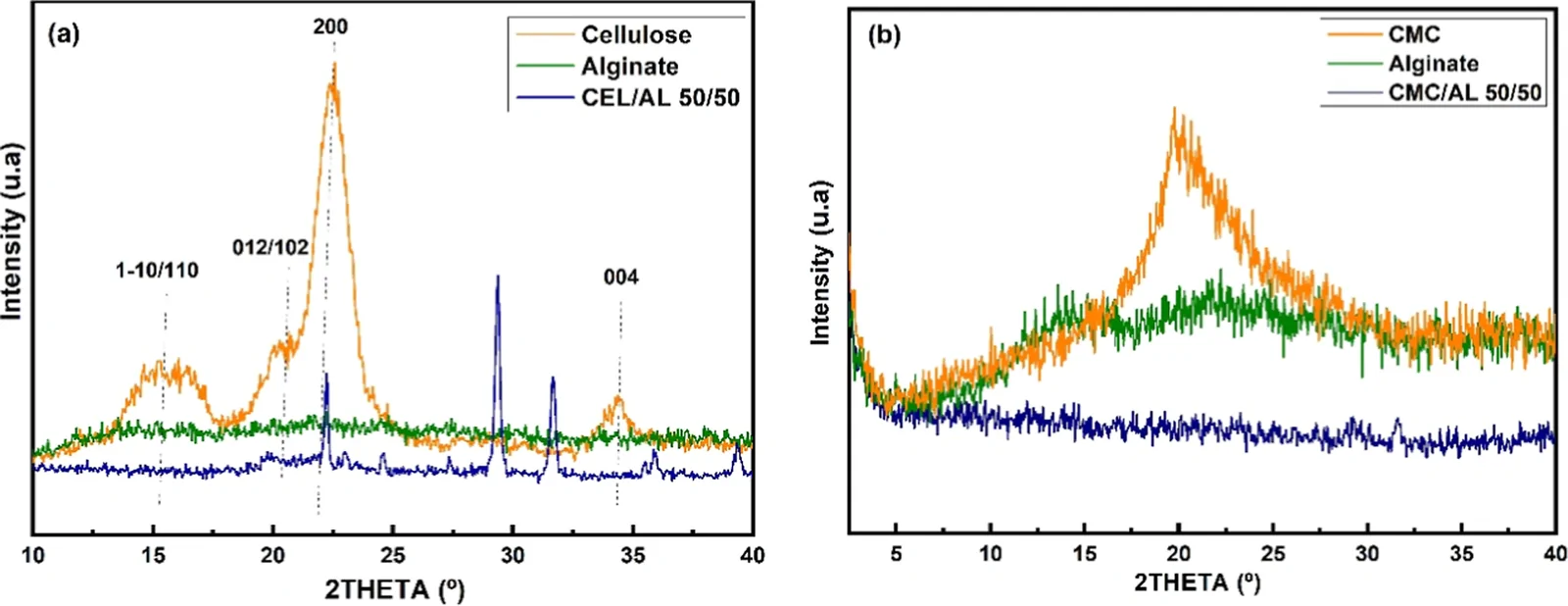
X-ray diffraction patterns for (a) cellulose, alginate and CEL/AL 50/50 hydrogel and (b) CMC, alginate and CMC/AL 50/50 hydrogel.

In the diffractogram presented in Figure 6a, it is possible to note that microcrystalline cellulose exhibits four characteristic peaks at 2θ = 15.6°, 20.2°, 22.4°, and 34.4°, corresponding to the Miller indices (11̅0/110), (012/102), (200), and (040), respectively. The main intensity contributors are the three Miller indices (11̅0), (110), and (200), which are characteristic of type I cellulose.^54,55^ For the CEL/AL 50/50 hydrogel (Figure 6a), two diffraction peaks are observed at 2θ ≈ 19.8° and 22°, corresponding to cellulose reflections; however, these peaks exhibit significantly reduced intensity compared to microcrystalline cellulose. Additionally, the absence of characteristic peaks at 2θ = 15.6° and 34.4° indicates a partial disruption of the native crystalline structure of cellulose during the dissolution and hydrogel formation processes.^56^ This reduction in crystallinity suggests increased chain disorder and reduced packing efficiency within the regenerated network. Additional weak diffraction features observed at approximately 2θ = 25°, 29.3°, 31.7°, 36°, and 39° may be associated with residual calcium species remaining in the hydrogel after cross-linking with CaCl_2_.^46,47^ However, as these features could not be unequivocally assigned based on the present XRD data, they were not considered in the interpretation of the cellulose crystalline phase.^57,58^

In contrast, CMC exhibits a broad diffraction peak around 2θ = 19.8°, which is indicative of its predominantly amorphous structure and low crystallinity.^59^ The XRD pattern of the CMC/AL 50/50 hydrogel (Figure 6b) shows no well-defined diffraction peaks, confirming the amorphous nature of the material. This structural organization is consistent with the enhanced swelling behavior and higher hydration enthalpy observed for this system (Figures 1b and 4b), as amorphous regions facilitate water diffusion and polymer chain mobility. Conversely, crystalline domains act as barriers to water penetration, limiting swelling capacity.^35^

### 3.6 Sorption Study of Methylene Blue (MB) Dye and Cd^2+^

Initial batch experiments were conducted to assess the potential use of CEL/AL 50/50 and CMC/AL 50/50 hydrogels as adsorbents for removing methylene blue (MB) dye and cadmium (Cd^2+^).

These contaminants are of significant environmental and health concern. The effects of adsorbent dosage, solution pH (Tables S4 and S5 Supporting Information), and contact time were investigated to determine the removal efficiency (ER %) and adsorption capacity (*q*_e_).

The present adsorption experiments were conducted under single-contaminant conditions; therefore, the observed sorption behavior should not be directly extrapolated to multicomponent water matrices. Because both adsorbates are cationic, the specific contribution of COO^–^-mediated electrostatic interactions cannot be independently resolved from the present experiments; comparison with a nonionic adsorbate would provide further insight into this contribution.

#### 3.6.1 Effect of Contact Time and Adsorption Kinetics

The adsorption profiles of MB and Cd^2+^ onto CEL/AL 50/50 and CMC/AL 50/50 hydrogels are presented in Figure 7. For MB dye, equilibrium was reached after 180 min for the CEL/AL 50/50 hydrogel, achieving a removal efficiency of 76.61%, whereas for CMC/AL 50/50 equilibrium was attained after 300 min, with a lower removal efficiency of 63.92% (Figure 7a,b).

**Figure 7:**
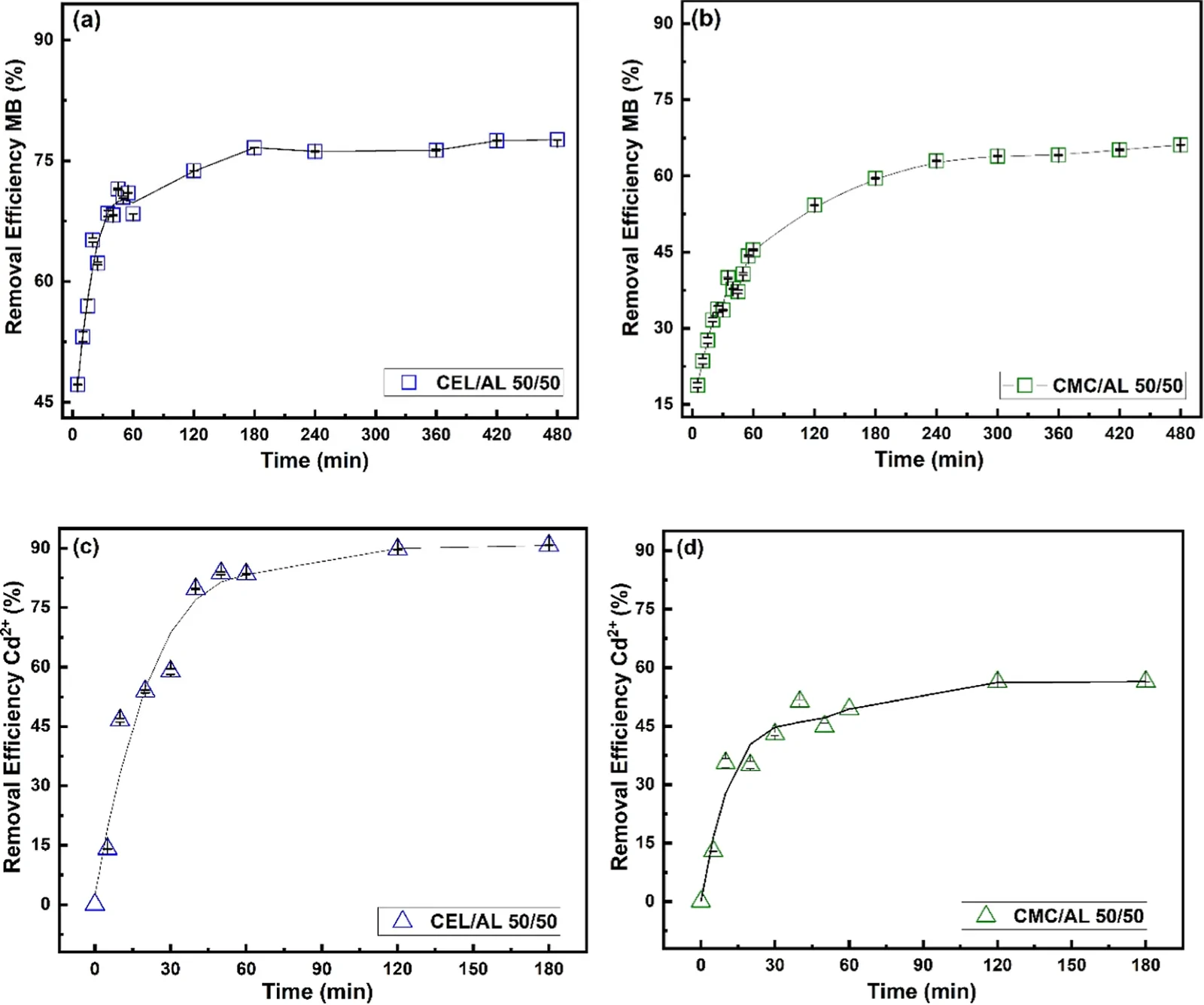
Removal efficiency (%) vs time at 25 °C for: MB dye 25 mg L^–1^ (5 to 480 min) 0.05 g of CEL/AL 50/50 (a) and CMC/AL 50/50 (b), natural solution pH (≅pH 8). Cd^2+^ ions 10 mg L^–1^ (5 to 180 min), 0.5 g of CEL/AL 50/50 (c) and 0.4 g of CMC/AL 50/50 (d), natural solution pH (pH ≅ 5.9) and final solution pH 9–10. Data are presented as mean values (*n* = 3); error bars represent standard deviation (SD).

For Cd^2+^ ions, equilibrium was achieved more rapidly, within 120 min, with removal efficiencies of 89% for CEL/AL 50/50 and 56% for CMC/AL 50/50 (Figure 7c,d). The faster equilibrium observed for Cd^2+^ suggests a stronger or more accessible interaction between the hydrogel functional groups and metal ions compared to MB molecules.

The initial stage of adsorption is characterized by a rapid increase in uptake, which can be attributed to the high availability of active sites on the hydrogel surface. As contact time increases, the adsorption rate decreases due to progressive occupation of these sites, leading to saturation and eventual equilibrium. An association between swelling behavior and adsorption kinetics is observed, particularly for MB. For MB adsorption, the equilibrium time is consistent with the swelling profiles, where maximum swelling occurs at approximately 60 min for CEL-based hydrogels and 180 min for CMC-based systems. In contrast, for Cd^2+^, maximum removal is achieved before the hydrogels reach their maximum swelling state, suggesting that adsorption is not solely governed by swelling but also by specific interactions between functional groups (e.g., COO^–^) and metal ions.

To further elucidate the adsorption mechanism, kinetic models including pseudo-first order (PFO), pseudo-second order (PSO), and intraparticle diffusion (DF) were applied to the experimental data.^22–24^

The adsorption kinetics were quantitatively evaluated by comparing the determination coefficients (*R* ^2^), the agreement between experimental and calculated adsorption capacities (*q*_e_), and the root-mean-square error (RMSE). The fitting results for the pseudo-first order (PFO), pseudo-second order (PSO), and intraparticle diffusion (DF) models are presented in Figure 8 and Tables S6 and S7 (Supporting Information).

**Figure 8:**
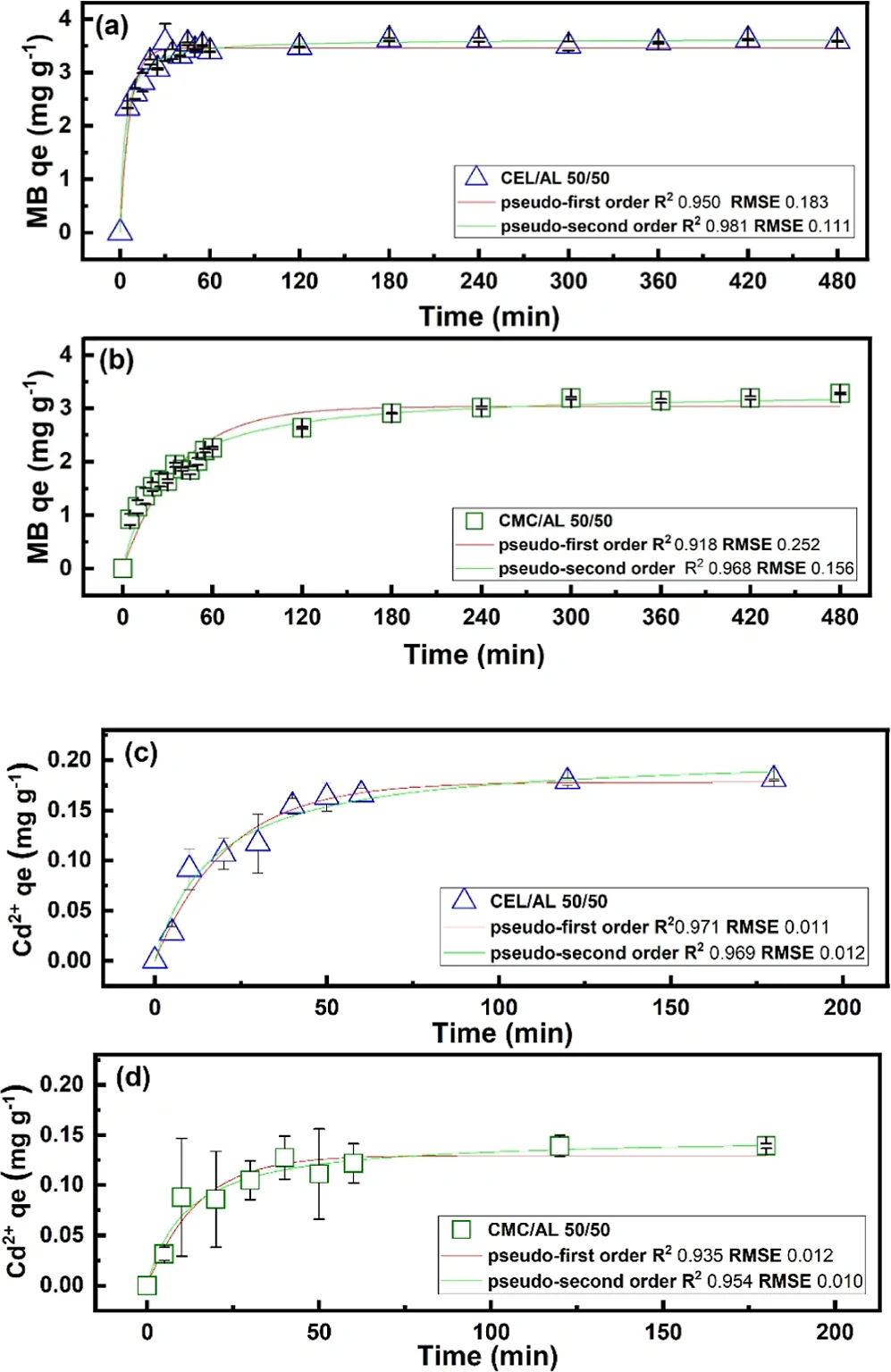
Nonlinear kinetic models of PFO, PSO at 25 °C for: MB dye 25 mg L^–1^ (5 to 480 min), 0.5 g of (a) CEL/AL 50/50 and (b) CMC/AL 50/50, natural solution pH ≅ pH 8 and final solution pH 9–10. Cd^2+^ 10 mg L^–1^ (5 to 180 min), 0.5 g of (c) CEL/AL 50/50 and 0.4 g of (d) CMC/AL 50/50, natural solution pH ≅ pH 5.9. Data are presented as mean values (*n* = 3); error bars represent standard deviation (SD).

As shown in Figure 8a,b,d, the PSO model provides the best fit for the adsorption of MB dye in both CEL/AL 50/50 and CMC/AL 50/50 hydrogels and for Cd^2+^ adsorption in CMC/AL 50/50 hydrogel. Whereas the PFO model better described Cd^2+^ adsorption in CEL/AL. These differences describe adsorption-rate behavior and do not, by themselves, establish molecular adsorption mechanisms.^60^

This behavior is consistent with literature reports, in which the PSO model is frequently associated with the adsorption of cationic dyes such as methylene blue, due to electrostatic interactions between negatively charged functional groups in the adsorbent and positively charged dye molecules.^60–63^

In contrast, the adsorption of Cd^2+^ ions onto the CEL/AL 50/50 hydrogel is better described by the PFO model (Figure 8c and Table S7), indicating that the adsorption rate is influenced by the availability of free active sites.^24^ This difference in kinetic behavior suggests that the adsorption mechanism depends not only on the nature of the adsorbate, but also on the structural and chemical characteristics of the hydrogel. In CEL/AL systems, the lower density of ionizable groups and the more compact network may limit strong interactions with Cd^2+^, favoring a process controlled by site availability rather than by stronger surface interactions.

The intraparticle diffusion model proposed by Weber and Morris (1963)^24^ as applied to evaluate the contribution of diffusion mechanisms to the adsorption process. The *q*_*t*_ versus *t* ^0.5^ profiles were used to qualitatively evaluate diffusion-related contributions to the adsorption kinetics.

For both CEL/AL 50/50 and CMC/AL 50/50 hydrogels, the adsorption of MB and Cd^2+^ occurs through a multi-stage process, as shown in Figure 9a–d and Tables S6 and S7 (Supporting Information). The first stage, observed within the first 30 min, is characterized by a steep slope and may be associated with the rapid diffusion of adsorbate species to the external surface of the hydrogels. This behavior reflects the high availability of active sites and strong interactions between the adsorbates and functional groups (COO^–^ and −OH) present in the hydrogel structure.^24,64^

**Figure 9:**
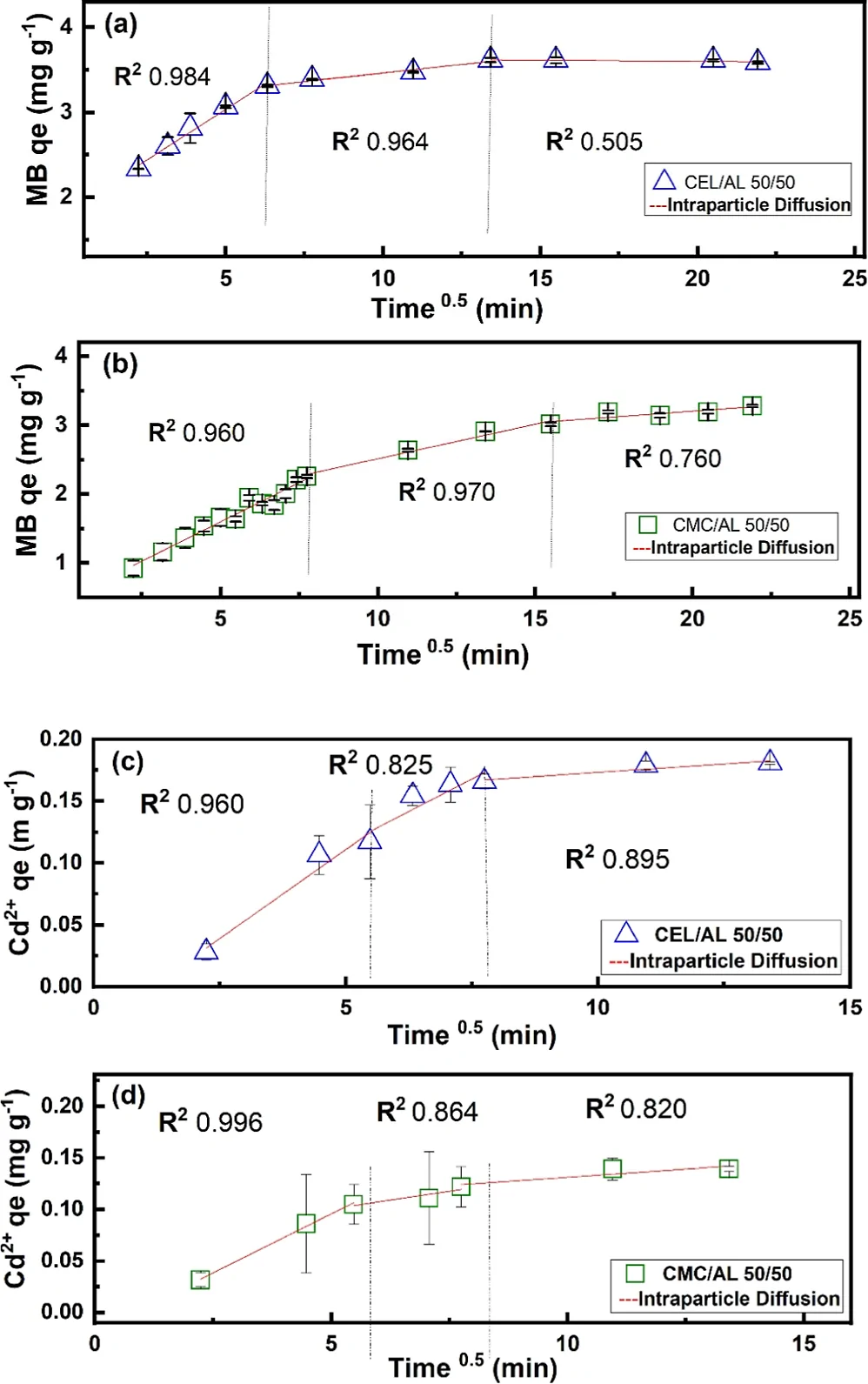
Intraparticle diffusion model (DF) at 25 °C for: MB dye 25 mg L^–1^ (5 to 480 min), 0.5 g of (a) CEL/AL 50/50 and (b) CMC/AL 50/50, natural solution pH ≅ pH 8 and final solution pH 9–10. Cd^2+^ 10 mg L^–1^ (5 to 180 min), 0.5 g of (c) CEL/AL 50/50 and 0.4 g of (d) CMC/AL 50/50, natural solution pH ≅ 5.9 and final solution pH 9–10. Data are presented as mean values (*n* = 3); error bars represent standard deviation (SD).

The intraparticle diffusion rate constants (*k*_1_) are lower for CMC/AL 50/50 compared to CEL/AL 50/50 for both contaminants, suggesting a slower diffusion process in the more compact CMC-based network. The intercept constant (*C*_df_), related to boundary layer effects, provides additional information on the diffusion behavior. For Cd^2+^, *C*_df_ values close to zero suggest a lower contribution of external mass transfer resistance, whereas the nonzero *C*_df_ values observed for MB adsorption suggest a contribution of boundary layer effects.^24^

The second stage, observed after approximately 30–60 min, corresponds to a gradual diffusion of the adsorbate into the internal structure of the hydrogels, with a reduced adsorption rate due to partial saturation of active sites. The third stage may be associated with the approach to equilibrium, occurring at approximately 480 min for MB and 180 min for Cd^2+^, where the adsorption rate becomes significantly lower, as reflected by reduced *k*_df_ values (Tables S6 and S7).^65^ These regions are interpreted as qualitative indications of different transport regimes rather than evidence that intraparticle diffusion is the sole rate-controlling mechanism.

A clear relationship between swelling behavior and adsorption mechanism can be observed. For MB, the equilibrium time coincides with the swelling profiles (60 min for CEL/AL and 180 min for CMC/AL), indicating that adsorption is strongly influenced by hydrogel expansion and adsorbate transport within the hydrated network. In contrast, Cd^2+^ adsorption reaches maximum removal efficiency before the hydrogels attain their maximum swelling. This suggests that Cd^2+^ adsorption is dominated by rapid interactions with readily accessible surface sites during the initial stage, as evidenced by the steep initial diffusion region in Figure 9.

After this initial stage, further swelling does not significantly enhance Cd^2+^ uptake, as the most accessible adsorption sites are already occupied. Conversely, MB adsorption is more dependent on diffusion into the internal structure of the hydrogel, making it more sensitive to the degree of swelling. The multilinear Weber–Morris plots are interpreted as qualitative indications of different transport regimes.

#### 3.6.2 Adsorption Isotherms

The CEL/AL 50/50 and CMC/AL 50/50 hydrogels exhibited removal efficiencies of 71% and 67% for MB dye (Figure 10a), and 98% and 72% for Cd^2+^ ions (Figure 10b), respectively. The higher removal efficiency observed for CEL/AL, particularly in Cd^2+^ adsorption indicates that the higher carboxylate content of CMC/AL alone does not determine adsorption performance and that both chemical functionality and network organization may contribute to the observed behavior.

**Figure 10:**
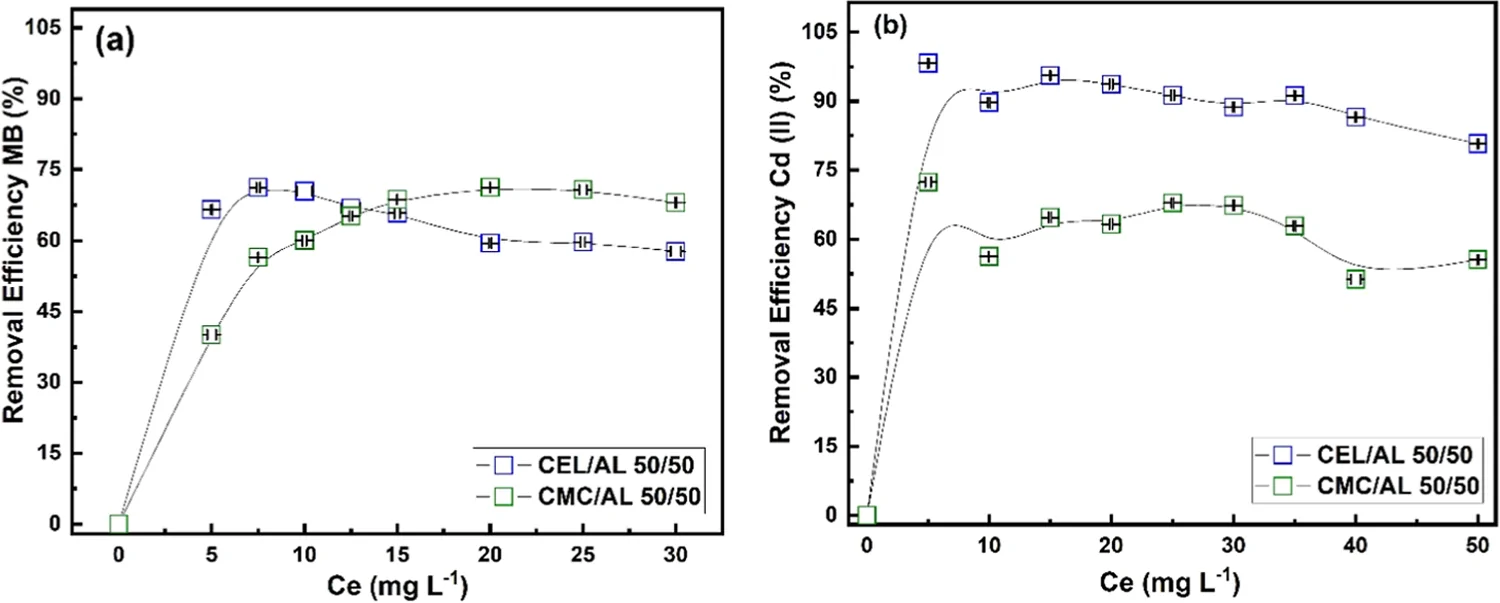
Removal efficiency (%) in 8 h at 25 °C at different concentrations of (a) MB dye (5 to 30 mg L^–1^), 0.05 g of the hydrogels, natural pH of the solution ≅pH 8 and final solution pH 9–10; (b) Cd^2+^ (5 to 50 mg L^–1^) 0.5 g of CEL/AL 50/50 and 0.4 g of CMC/AL 50/50, natural pH of the solution ≅pH 5.9. And final solution pH 9–10. * The results are expressed in (mean ± standard error).

At lower initial concentrations of MB and Cd^2+^, a large number of available adsorption sites relative to the amount of adsorbate results in higher removal efficiencies. As the initial concentration increases, the progressive occupation of active sites leads to increased competition among adsorbate species, reducing the overall removal efficiency until equilibrium is reached.^66,67^

To further clarify the adsorption mechanism of MB dye and Cd^2+^ by CEL/AL 50/50 and CMC/AL 50/50 hydrogels, equilibrium data at different concentrations were fitted using nonlinear models (Langmuir, Freundlich and Sips) and the linear Dubinin–Radushkevich (DR) model, Figure 11.^68^

**Figure 11:**
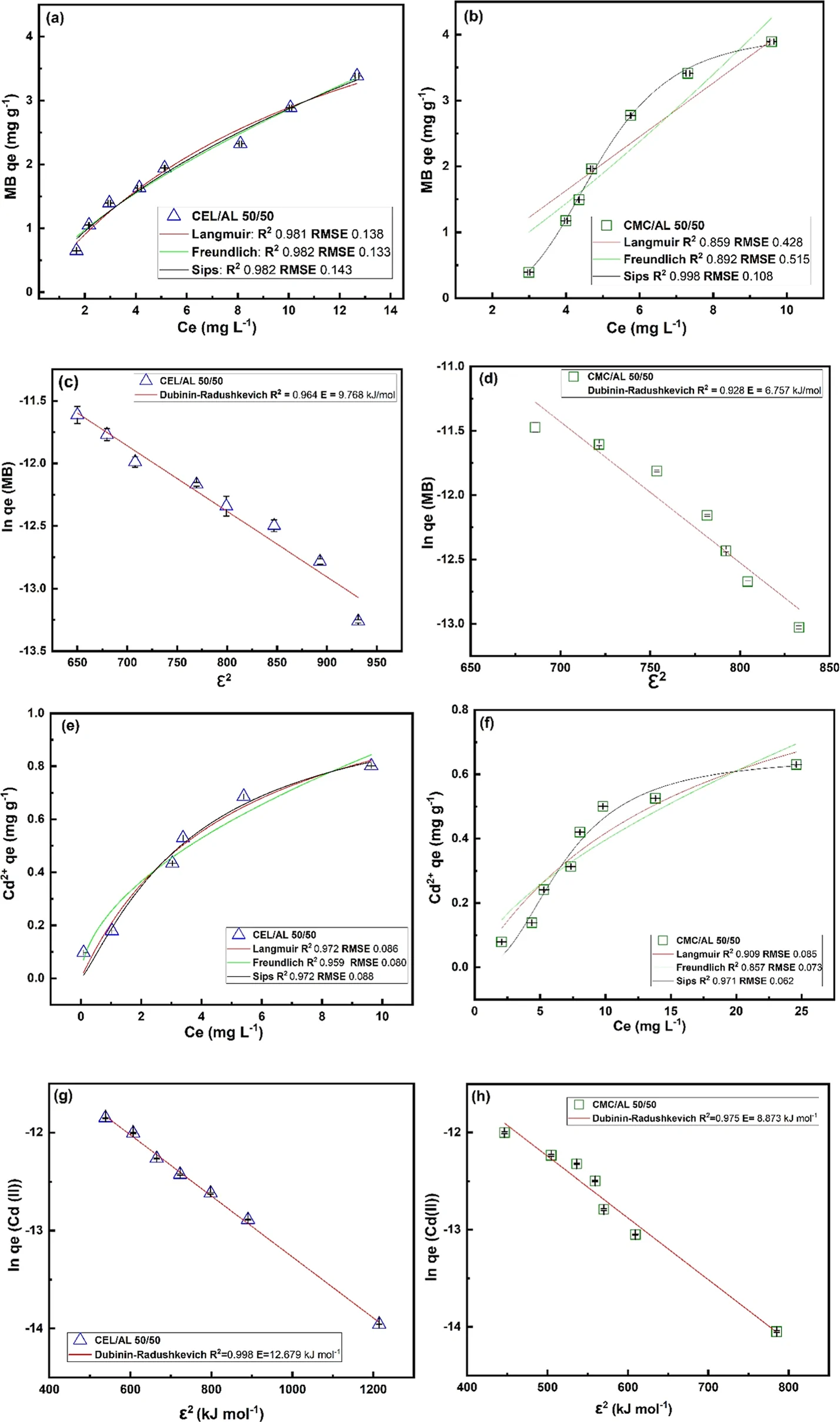
Langmuir, Freundlich and Sips adsorption isotherms for MB dye (5–30 mg L^–1^) using 0.05 g of hydrogels, initial solution pH ≅ 8 and final solution pH 9–10, 25 °C: (a) CEL/AL 50/50, (b) CMC/AL 50/50 and linear model Dubinin–Radushkevich (DR) for (c) CEL/AL 50/50 and (d) CMC/AL 50/50. Langmuir, Freundlich and Sips adsorption isotherms for Cd^2+^ (5–50 mg L^–1^) using 0.5 g of CEL/AL 50/50 and 0.4 g of CMC/AL 50/50, initial solution pH ≅ 5.9 and final solution pH 9–10: (e) CEL/AL 50/50, (f) CMC/AL 50/50 and linear DR model for (g) CEL/AL 50/50 and (h) CMC/AL 50/50. Data are presented as mean values (*n* = 3); error bars represent standard deviation (SD).

For the CEL/AL 50/50 hydrogel, MB adsorption is best described by the Freundlich model, as evidenced by higher *R* ^2^ values and lower RMSE (Figure 11a and Table S8). This behavior indicates a heterogeneous surface with adsorption occurring in multilayers, reflecting a non-uniform distribution of adsorption sites with different energies.^65,69^ In contrast, both Langmuir and Sips provided satisfactory fits to Cd^2+^ adsorption onto CEL/AL 50/50 (Table S9).

For the CMC/AL 50/50 hydrogel, both MB and Cd^2+^ adsorption are best described by the Sips model, as indicated by higher *R* ^2^ values, lower RMSE, and calculated *q*_m_ values consistent with experimental data.^68,70^

The adsorption mechanisms can be further interpreted based on the chemical structure of the hydrogels. For MB dye, adsorption may involve electrostatic interactions between the cationic dye and negatively charged carboxylate groups (COO^–^) present in alginate and CMC. Additionally, n–π interactions between oxygen-containing groups in the hydrogel (electron donors) and the aromatic rings of MB (electron acceptors) may contribute to the adsorption process.^63,71^ These interactions are consistent with literature reports highlighting the affinity of carboxymethyl cellulose for cationic dyes.^72^

The adsorption of cationic species on the surface of polymeric hydrogels can be determined by the interactions between the adsorbate and the hydrogels, e.g., electrostatic, van der Waals, and hydrogen interactions.^73,74^ Ion exchange also plays a key role in the adsorption process of metal ions such as Cd^2+^.^11^

The influence of surface charge on adsorption was evaluated through the point of zero charge (pH_PZC_). The CEL/AL 50/50 and CMC/AL 50/50 hydrogels exhibited pH_PZC_ values of 7.56 and 8.15, respectively (Table S10). However, the weak dependence of MB adsorption on pH indicates that surface charge alone does not determine its adsorption behavior. At pH values above pH_PZC_, the hydrogel surface becomes negatively charged, which may favor interactions with of cationic species such as MB and Cd^2+^.^66^

For MB adsorption, the initial solution pH (∼8) is close to the pH_PZC_ values of both hydrogels. The increase in pH to values between 9 and 10 during equilibrium suggests the release of OH^–^ ions, possibly associated with ion exchange processes.^46^

For Cd^2+^, the initial pH (∼5.9) is below the pH_PZC_, which is initially unfavorable for adsorption. However, the increase in pH to values between 9 and 10 after contact with the hydrogels indicates deprotonation of functional groups and possible formation of coordination complexes between Cd^2+^ and carboxylate groups, enhancing adsorption capacity.^11^ This behavior is reflected in the high removal efficiencies observed for Cd^2+^.

The DR isotherm model was used to compare the model-derived mean adsorption energies. For CEL/AL 50/50, the calculated mean free energy (*E*) values for both MB and Cd^2+^ are greater than 8 kJ mol^–1^ (Figure 11c,g). In contrast, for CMC/AL 50/50, *E* values are ≤8 kJ mol^–1^ (Figure 11d,h).^60,62^ These model-derived *E* provide comparative information among the adsorption systems investigated in this study.

## 4 Conclusions

This study demonstrates that the physicochemical and functional behavior of CEL/AL 50/50 and CMC/AL 50/50 hydrogels reflects the combined contributions of chemical functionality and network organization.

Structural analyses revealed distinct organization patterns in the two systems. The CEL/AL hydrogel retained partial cellulose crystallinity, indicating local ordering, despite exhibiting a porous and heterogeneous network at the microscale, whereas the CMC/AL hydrogel exhibited a predominantly amorphous structure associated with enhanced chain flexibility and higher hydration capacity. These physicochemical differences were associated with distinct adsorption responses. Adsorption of the 50/50 formulations enabled direct comparison of the sorption behavior of CEL/AL and CMC/AL hydrogels at the same nominal polymer ratio. Their physicochemical behavior reflects differences in both chemical functionality and network organization, while the kinetic and equilibrium models provide complementary information on the rate and equilibrium behavior under the investigated conditions. The Dubinin–Radushkevich (DR) model further indicated differences in the model-derived adsorption energies of CEL/AL and CMC/AL.

Complementary FTIR and thermal analyses confirmed the presence of significant polymer–polymer interactions and network rearrangements induced by hydrogel formation. Changes in characteristic absorption bands and thermal transitions provided molecular-level evidence of structural reorganization, supporting the observed differences in sorption behavior.

Overall, this work advances a structure-driven perspective on polysaccharide hydrogels, indicating that adsorption behavior reflects contributions from network hydration and adsorbate transport in addition to the dry-state textural properties. By employing adsorption as a mechanistic probe, this study deepens the understanding of structure–property relationships in CEL/AL and CMC/AL systems and supports their development as sustainable materials for water treatment applications.

## Supplementary Material



## Notes

^1^H NMR spectrum and Gaussian deconvolution data for sodium alginate, including the determination of its structural parameters and *M*/*G* ratio; calibration curves for the quantification of methylene blue (MB) and Cd^2+^; O–H stretching band positions and wavenumber shifts of CEL/AL and CMC/AL hydrogels and their respective neat polymer components; peak temperature and enthalpy of the first thermal event obtained from DSC analysis of the neat polymers and CEL/AL and CMC/AL hydrogels; removal efficiency (ER %) of MB and Cd^2+^ at different hydrogel masses and pH values; nonlinear kinetic parameters obtained from the pseudo-first-order, pseudo-second-order, and linear intraparticle diffusion models for MB and Cd^2+^; calculated coefficients from adsorption isotherm models for MB and Cd^2+^; pH at the point of zero charge (pH_PZC_) values of CEL/AL and CMC/AL hydrogels (PDF)
