# Supplementary material for: Decoupling Network Architecture and Sorption Mechanisms in Cellulose–Alginate and Carboxymethylcellulose–Alginate Hydrogels
Source: ACS Omega. 2026 Oct 3;11(41):63877–92. doi: 10.1021/acsomega.6c07886 (PMC13689206; doi:10.1021/acsomega.6c07886)
Supplement: Supplementary file 1 [file ao6c07886_si_001.pdf]

Decoupling Network Architecture and Sorption Mechanisms in Cellulose–Alginate and CMC–Alginate Hydrogels

Paula Paulino Silva[a], Vinicius Augusto de Melo Gomes[a], Juliano Brisola[a], Francisnara Tonholi[a], Gizilene Maria de Carvalho[a*]*

[a]Department of Chemistry, State University of Londrina, Rodovia Celso Garcia Cid,| Pr 445 Km 380 | Campus Universitário Londrina, 86057-970, Paraná, BR.

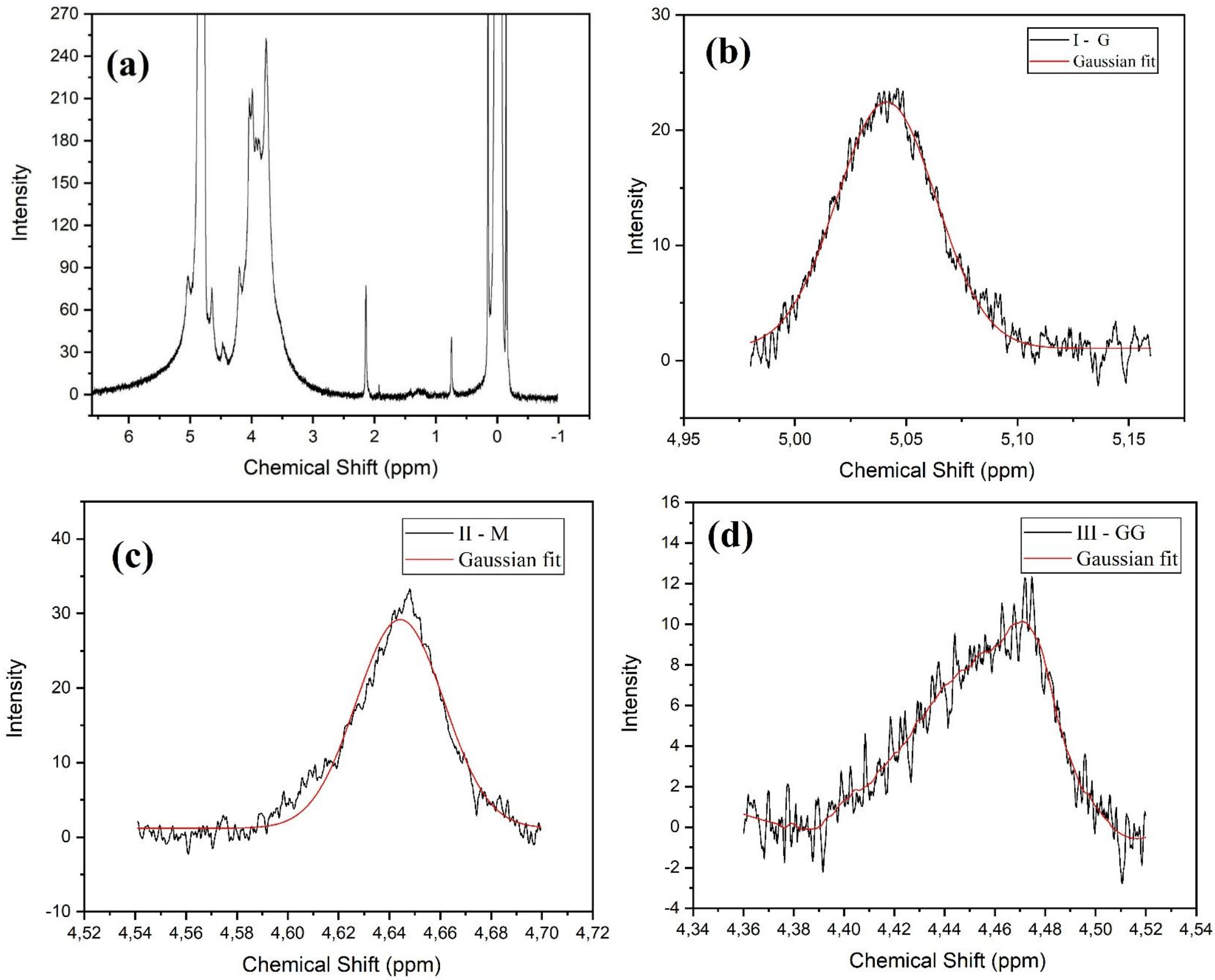

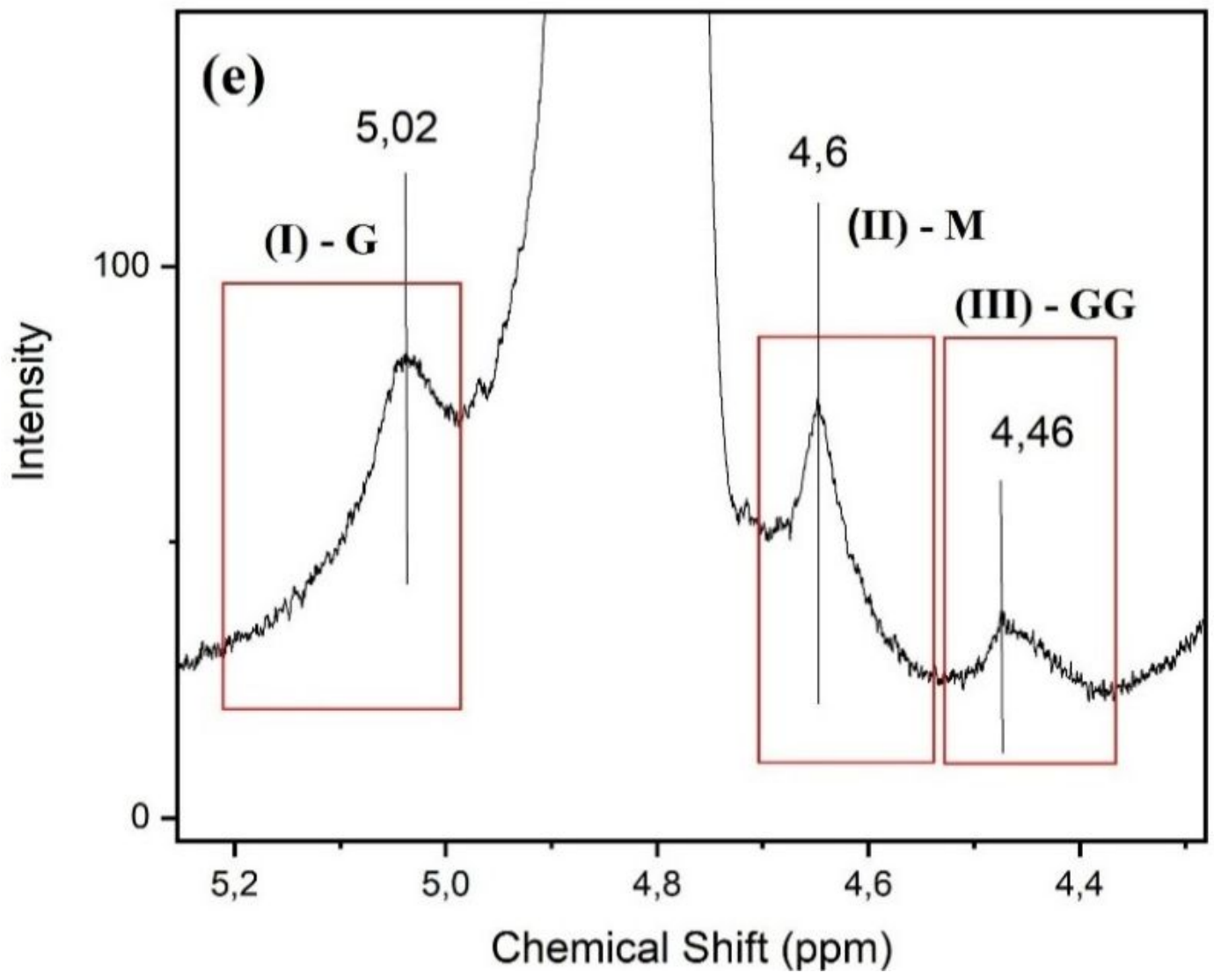

Figure S1. ^{1}H–NMR spectrum and Gaussian deconvolution procedure used for determination of alginate structural parameters. (a) Complete ^{1}H–NMR spectrum of sodium alginate and expanded spectral regions used for peak fitting: (b) Region I (G residues; 5,2–4,9 ppm), (c) Region II (M residues; 4.8–4.6 ppm), and (d) Region III (GG sequences; 4.6–4.4 ppm). (e) Selected ^{1}H–NMR spectral regions used for the determination of the mannuronic acid (M) to guluronic acid (G) ratio of sodium alginate. Gaussian fitting was applied to resolve partially overlapped signals and improve peak area integration for calculation of FG, FM, and M/G ratio.

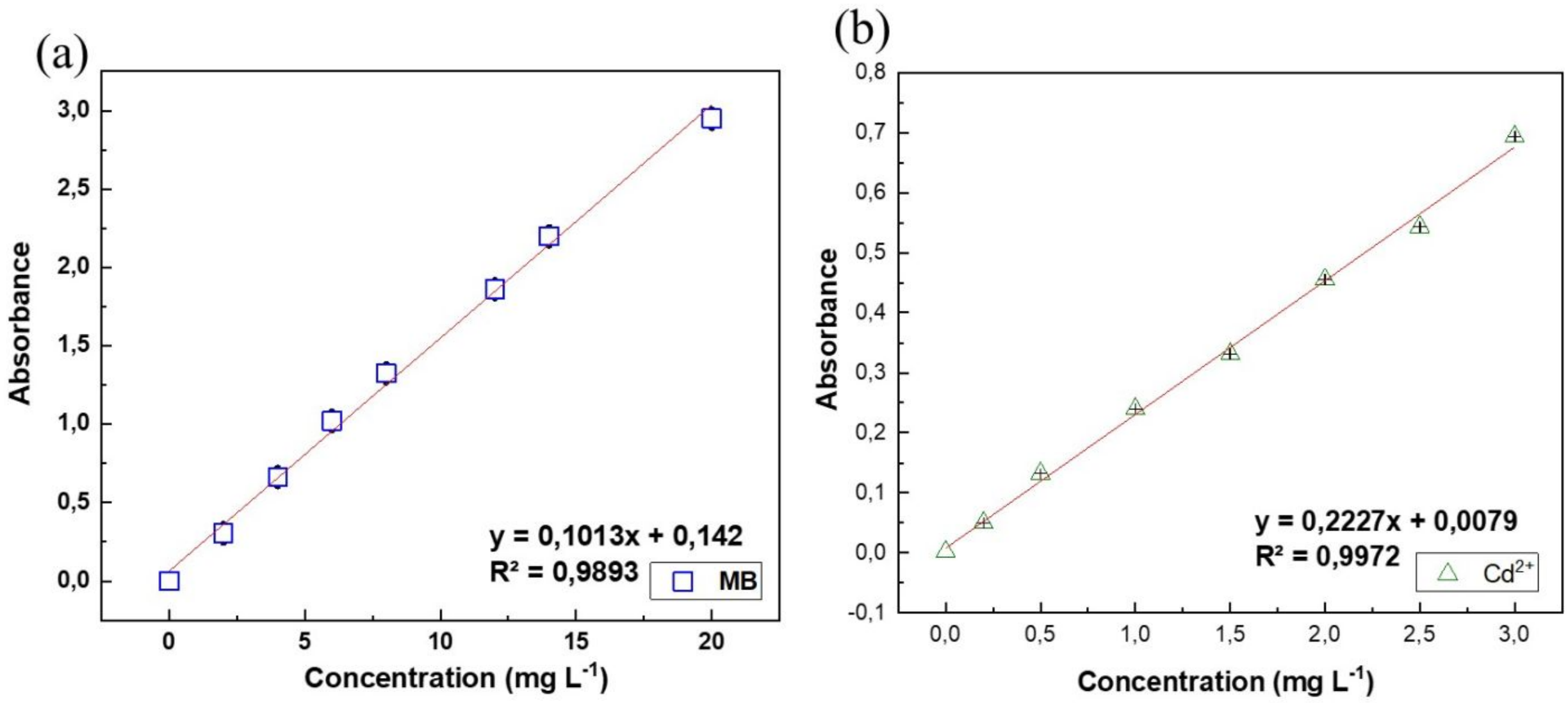

Figure S2. Calibration curves used for adsorbate quantification: (a) methylene blue (MB) measured at 665 nm and (b) Cd^{2+} in the concentration range of 0.2–3.0 mg L^{-1}. The solid lines represent the corresponding linear regressions.

Table S1. O-H stretching band positions and wavenumber shifts of CEL/AL and CMC/AL hydrogels and their neat polymer components.

Sample	ν_{max} of O-H (cm^{-1})	ν_{max} vs reference polymer (cm^{-1})
**CMC/AL hydrogels**		
**AL**	3223	—
**CMC**	3243	0.0
**CMC/AL 25/75**	3344	+101
**CMC/AL 40/60**	3337	+94
**CMC/AL 50/50**	3374	+131
**CEL/AL hydrogels**		
**CEL**	3330	—
**CEL/AL 75/25**	3339	+9
**CEL/AL 60/40**	3351	+21
**CEL/AL 50/50**	3337	+7
**CEL/AL 40/60**	3337	+7
**CEL/AL 25/75**	3347	+17
**AL**	3223	—

* vmax was determined by local maximum analysis of the O-H stretching region and verified by the corresponding zero-crossing of the first derivative. When multiple zero-crossings were observed, the second derivative was used to support assignment of the relevant band maximum, while minor derivative features were disregarded. The wavenumber shift (Δv) was calculated relative to the corresponding neat cellulose-based component (CEL or CMC) Positive Δv values indicate shifts toward higher wavenumbers.

Calculation: $\Delta \nu = \nu_{max,hydrogel} - \nu_{max,polymer}$.

Table S2. Peak temperature and enthalpy of the first thermal event observed in the DSC curves of the neat polymers and CEL/AL and CMC/AL hydrogels.

Sample	Tpeak (°C),	ΔH J/g	Tpeak (°C)
**AL**	98	1680	253
**CEL**	80	139	338
**CMC**	102	2220	318
**CEL/AL 25/75**	82	251	216
**CEL/AL 40/60**	87	209	226
**CEL/AL 50/50**	88	272	208
**CEL/AL 60/40**	82	126	222
**CEL/AL 75/25**	79	149	222
**CMC/AL 25/75**	97	187	210
**CMC/AL 40/60**	96	286	215
**CMC/AL 50/50**	102	352	222

*Tpeak corresponds to the temperature at the maximum of the first DSC thermal event; ΔH corresponds to the enthalpy associated with this event.

Table S3. Surface areas calculated by BET for CEL/AL and CMC/AL hydrogels.

**Hydrogels CEL/AL**	**Specific surface area (m^2/g) (BET)**	**Specific pore volume (cm^3/g) (BET)**	**Average Pore Size (nm) (BET)**
**25/75**	0.31	$1.9x10^{-3}$	12.33
**40/60**	0.57	$3.3x10^{-3}$	11.73
**50/50**	0.40	$1.7x10^{-3}$	8.74
**60/40**	0.16	$0.5x10^{-3}$	5.81
**75/25**	0.12	$0.3x10^{-3}$	4.41
**Hydrogels CMC/AL**	Specific surface area (m^2/g) (BET)	Specific pore volume (cm^3/g) (BET)	Average Pore Size (nm) (BET)
**25/75**	0.04	$1.3x10^{-4}$	5.74
**40/60**	0.05	$0.6x10^{-4}$	2.83
**50/50**	0.09	$2.1x10^{-4}$	5.03

Table S4. Removal efficiency (ER%) of MB dye and Cd^{2+} ions in different hydrogel masses of CEL/AL 50/50 and CMC/AL 50/50 with initial solution concentration of 25 mg L^{-1} of MB and 10 mg L^{-1} of Cd^{2+} ions in eight hours with natural pH of the solution (pH ≅ 8.0 MB and pH ≅ 5.9 Cd^{2+}) at 25ºC.

**Amount in grams CEL/AL 50/50**	**ER% MB**	**ER% Cd^{2+}**
**0.05**	33.58^{a}±2.16	-
**0.10**	43.67^{a}±2.64	73.13^{e}± 0.52
**0.20**	-	82.36^{d}±0.73
**0.30**	-	88.41^{c}±0.94
**0.40**	-	91.34^{ab}±0.57
**0.50**	-	92.75^{a}±0.82
**0.60**	-	89.40^{bc}±1.30
**0.70**	-	90.70^{abc}±0.66
**Amount in grams CMC/AL 50/50**	**ER% MB**	**ER%** Cd^{2+}
**0.05**	50.14^{a}±1.67	-
**0.10**	57.90^{a}±0.70	47.85^{b}±2.36
**0.20**	-	54.50^{ab}±2.00
**0.30**	-	56.54^{a}±2.48
**0.40**	-	60.24^{a}±2.34
**0.50**	-	56.52^{a}±0.03

* Results are expressed as (mean ± standard deviation). Different letters in the same column indicate significant differences (P < 0.05) according to Tukey's test.

Table S5. Removal efficiency (ER%) at different pH of MB dye 25 mg L^{-1} with 0.05 g of CEL/AL 50/50 and CMC/AL 50/50 hydrogel and Cd^{2+} ions 10 mg L^{-1} with 0.5 g of CEL/AL 50/50 hydrogel and 0.4 g of CMC/AL 50/50 hydrogel, in eight hours at room temperature (≅ 25 ºC)

pH Initial	CEL/AL 50/50 ER MB%	CMC/AL 50/50 ER MB%	CEL/AL 50/50 ER (%) Cd^{2+}	CMC/AL 50/50 ER (%) Cd^{2+}
**4.0**	80.40^{a}±5.07	72.30^{cd}±0.20	92.27^{a}±1.76	56.70^{a}±3.05
**5.0**	73.88^{a}±3.66	72.09^{d}±0.63	91.60^{a}±0.58	50.11^{a}±3.36
**6.0**	79.21^{a}±0.77	73.86^{bc}±0.09	90.49^{a}±0.41	51.46^{a}±2.91
**7.0**	78.54^{a}±0.98	72.17^{cd}±0.01	91.63^{a}±0.26	54.45^{a}±0.21
**8.0**	79.78^{a}±0.92	74.78^{b}±0.22	91.43^{a}±1.70	51.76^{a}±2.60
**9.0**	80.42^{a}±0.12	74.84^{b}±0.29	91.27^{a}±0.24	52.51^{a}±3.52
**10.0**	80.30^{a}	76.65^{a}±0.32	90.28^{a}±0.02	51.91^{a}±0.97

* Results are expressed as (mean ± standard deviation). Different letters in the same column indicate significant differences ($P < 0.05$) according to Tukey's test.

Table S6. Nonlinear kinetic parameters of pseudo–first order (PFO), pseudo–second order (PSO) and intraparticle diffusion (DF) at different contact times with MB dye 25 mg L^{-1} at natural pH of the solution pH ≅ 8 and final pH of the solution 9.0–10.0 at room temperature ≅ 25ºC for CEL/AL 50/50 and CMC/AL 50/50 hydrogels.

**Methylene Blue Dye Kinetics (MB) 25 mg L^{-1}**		
**Pseudo first order (PFO)**	**CEL/AL 50/50**	**CMC/AL 50/50**
$q_{e\,exp}$ **(mg g^{-1})**	3.615	3.277
q_e **(mg g^{-1})**	3.462	3.063
k_1 **(min^{-1})**	0.158	0.027
**R^2**	0.950	0.918
**RSS**	<0.001	<0.001
**RMSE**	0.183	0.252
**Pseudo second order (PSO)**	**CEL/AL 50/50**	**CMC/AL 50/50**
$q_{e\,exp}$ **(mg g^{-1})**	3.615	3.338
q_e **(mg g^{-1})**	3.629	3.338
k_2 **(g mg^{-1} min^{-1})**	0.088	0.011
**R^2**	0.981	0.968
**RSS**	<0.001	<0.001
**RMSE**	0.111	0.156
**Intraparticle Diffusion (DF)**	**CEL/AL 50/50**	**CMC/AL 50/50**
k_{df} **(mg $g^{-1}min^{-0,5}$)**	0.238	0.228
	0.041	0.100
	-0.002	0.033
**C_{df} (mg g^{-1})**	1.844	0.455
	3.055	1.509
	3.649	2.530
**R^2**	0.984	0.960
	0.964	0.970
	0.505	0.760
**RMSE**	0.048	0.080
	0.025	0.058

	0.012	0.055

*$q_{e\,exp}$: experimental equilibrium adsorption capacity (mg g^{-1}); k_1, k_2, k_{df}: rate constants for pseudo–first order, pseudo–second order sorption and intraparticle diffusion constant, respectively. q_e: equilibrium adsorption capacity estimated from the kinetic-model fit (mg g^{-1}) C_{df}: constant related to the resistance to diffusion in the boundary layer (mg g^{-1}). R^2: coefficient of determination, RSS: sum of squared errors. RMSE: Root means square error.

Table S7. Parameters obtained from pseudo–first order, nonlinear pseudo–second order kinetics and intraparticle diffusion at different contact times with Cd^{2+} ions 10 mg L^{-1} at natural pH of the solution pH ≅ 5.9 and final pH of the solution 9.0–10.0 at room temperature ≅ 25 ºC for CEL/AL 50/50 and CMC/AL 50/50 hydrogels.

kinetics Íons Cd^{2+} 10 mg L^{-1}		
**Pseudo first order (PFO)**	CEL/AL 50/50	CMC/AL 50/50
$q_{e\,exp}$ **(mg g^{-1})**	0.180	0.139
q_e **(mg g^{-1})**	0.178	0.129
k_1 **(min^{-1})**	0.047	0.0683
**R^2**	0.971	0.935
**RSS**	0.001	0.001
**RMSE**	0.011	0.012
**Pseudo second order (PSO)**	CEL/AL 50/50	CMC/AL 50/50
$q_{e\,exp}$ **(mg g^{-1})**	0.180	0.139
q_e **(mg g^{-1})**	0.206	0.148
k_2 **(g mg^{-1} min^{-1})**	0.282	0.586
**R^2**	0.969	0.954
**RSS**	0.001	<0.001
**RMSE**	0.012	0.010
**Intraparticle Diffusion (DF)**	**CEL/AL 50/50**	**CMC/AL 50/50**
k_{df} **(mg g^{-1} $min^{-0,5}$)**	0.028	0.022
	0.021	0.007
	0.003	0.003
**C_{df} (mg g^{-1})**	-0.033	-0.018
	0.010	0.065
	0.146	0.099
**R^2**	0.960	0.996
	0.825	0.864
	0.895	0.820
**RMSE**	0.011	0.003

	0.011	0.004
	0.004	0.006

*$q_{e\ exp}$: experimental equilibrium adsorption capacity (mg g^{-1}); k_1, k_2, k_{df}: rate constants for pseudo–first order, pseudo–second order sorption and intraparticle diffusion constant, respectively. q_e: equilibrium adsorption capacity estimated from the kinetic-model fit (mg g^{-1}) C_{df}: constant related to the resistance to diffusion in the boundary layer (mg g^{-1}). R^2: coefficient of determination, RSS: sum of squared errors. RMSE: Root means square error

Table S8. Calculated coefficients for the isothermal models of the adsorption capacity (q_e) of MB dye with 0.05 g of hydrogel at different initial concentrations of the MB dye solution in eight hours with natural pH of the solution ≅ pH 8.0 and final pH of the solution 9.0–10.0 at room temperature ≅ 25ºC.

**Isothermal Models of Adsorption Capacity (q_e) of dye MB**				
**Model**	Non–linear form	Parameters	CEL/AL 50/50	CMC/AL 50/50
**Freundlich**	$q_e = k_f * Ce^{1/n}$	q_{exp} (mg g^{-1})	4.327	3.894
		k_f (L $mg^{-1})^{1/n}$	0.613	0.257
		n	1.493	0.805
		R^2	0.982	0.892
		RSS	0.105	1.099
		RMSE	0.133	0.428
**Langmuir**	$q_e = \left(\frac{q_m * k_l * c_e}{1 + k_l * c_e}\right)$	q_m (mg g^{-1})	6.401	2.174 x 10^6
		q_{exp} (mg g^{-1})	4.327	3.894
		k_l (L mg^{-1})	0.082	1.878
		R_L	0.032	0.011
		R^2	0.981	0.859
		RSS	0.114	1.588
		RMSE	0.138	0.515
**Sips**	$q_e = \frac{q_m K_S C_e^{1/ns}}{1 + K_S C_e^{1/ns}}$	q_m(mg g^{-1})	14.47	4.118
		q_{exp} (mg g^{-1})	4.327	3.894
		n_s	0.781	4.528
		k_s	0.041	0.001
		R^2	0.982	0.998
		RSS	0.102	0.058
		RMSE	0.143	0.108

q_e: adsorption capacity at equilibrium (mg g^{-1}); q_m: maximum monolayer adsorption capacity (mg g^{-1}); qs: theoretical saturation capacity; C_e: equilibrium adsorbate concentration in the solution (mg L^{-1}); C_i: initial adsorbate concentration (mg L^{-1}); k_l: Langmuir constant related to the adsorbate–adsorbent affinity (L mg^{-1}); k_f: Freundlich constant (L mg^{-1}); n: Freundlich empiric parameter; k_S: Sips constant; n_S: Sips heterogeneity parameter; RL: Langmuir separation factor; β: D–R constant (mol^2 kJ^{-2}); ε: Polanyi potential (kJ^2 mol^{-2}); R: universal gas constant (8.314 J mol^{-1} K^{-1}) or 0.008314 kJ mol^{-1} K^{-1}; T: absolute temperature (K); and E: represents the average free energy of adsorption (kJ mol^{-1}). R^2: coefficient of determination, RSS: sum of squared errors. RMSE: Root means square error.

Table S9. Calculated coefficients for the isothermal models of the adsorption capacity (q_e) of the Cd^{2+} ion with 0.5 g of CEL/AL 50/50 hydrogel and 0.4 g of CMC/AL 50/50 hydrogel in different initial concentrations of the Cd^{2+} ion solution in eight hours with natural pH of the solution pH ≅ 5.9 and final pH of the solution 9.0–10.0 at room temperature ≅ 25ºC.

Isothermal Models of Adsorption Capacity (q_e) of Cd^{2+}				
**Model**	Non–linear form	Parameters	CEL/AL 50/50	CMC/AL 50/50
**Freundlich**	$q_e = k_f * Ce^{1/n}$	k_f (mg g^{-1})(mg L)$^{1/n}$	0.252	0.094
		n	1.879	1.600
		R^2	0.959	0.858
		RSS	0.015	0.038
		RMSE	0.080	0.085
**Langmuir**	$q_e = \left(\frac{q_m * k_l * c_e}{1 + k_l * c_e}\right)$	q_m (mg g^{-1})	1.256	1.142
		q_{exp} (mg g^{-1})	0.801	0.689
		k_l (L mg^{-1})	0.197	0.058
		R_L	0.014	0.019
		R^2	0.972	0.909
		RSS	0.011	0.024
		RMSE	0.086	0.073
**Sips**	$q_e = \frac{q_m K_S C_e^{1/ns}}{1 + K_S C_e^{1/ns}}$	q_m (mg g^{-1})	1.084	0.655
		q_{exp} (mg g^{-1})	0.801	0.689
		n_s	1.191	2.395
		k_{sips}	0.205	0.010
		R^2	0.972	0.970
		RSS	0.010	0.007
		RMSE	0.088	0.062

q_e: adsorption capacity at equilibrium (mg g^{-1}); q_m: maximum monolayer adsorption capacity (mg g^{-1}); qs: theoretical saturation capacity; C_e: equilibrium adsorbate concentration in the solution (mg L^{-1}); C_i: initial adsorbate concentration (mg L^{-1}); k_l: Langmuir constant related to the adsorbate–adsorbent affinity (L

mg⁻¹); k_f: Freundlich constant (L mg⁻¹); n: Freundlich empiric parameter; k_S: Sips constant; n_S: Sips heterogeneity parameter; RL: Langmuir separation factor; β: D–R constant ($mol^2\ kJ^{-2}$); ε: Polanyi potential ($kJ^2\ mol^{-2}$); R: universal gas constant (8.314 J $mol^{-1}\ K^{-1}$) or 0.008314 kJ $mol^{-1}\ K^{-1}$; T: absolute temperature (K); and E: represents the average free energy of adsorption (kJ mol^{-1}). R^2: coefficient of determination, RSS: sum of squared errors. RMSE: Root means square error.

Table S10. pH at the Point of Zero Charge (pHPCZ) for CEL/AL and CMC/AL hydrogels at room temperature ≅25ºC.

**Hydrogels CEL/AL**	**pH PCZ**
**25/75**	$7.98^{a}\pm0.58$
**40/60**	$8.33^{a}\pm0.82$
**50/50**	$7.56^{a}\pm0.36$
**60/40**	$7.39^{a}\pm0.14$
**75/25**	$7.67^{a}\pm0.24$
**Hydrogels CMC/AL**	pH (PCZ)
**25/75**	$5.15^{b}\pm0.35$
**40/60**	$6.20^{b}\pm0.10$
**50/50**	$8.15^{a}\pm0.35$

* Results are expressed as (mean ± standard deviation). Different letters in the same column indicate significant differences ($P < 0.05$) according to Tukey's test.
